# Mitochondrial Cristae Architecture and Functions: Lessons from Minimal Model Systems

**DOI:** 10.3390/membranes11070465

**Published:** 2021-06-23

**Authors:** Frédéric Joubert, Nicolas Puff

**Affiliations:** 1 Laboratoire Jean Perrin, CNRS, Sorbonne Université, UMR 8237, 75005 Paris, France; frederic.joubert@upmc.fr; 2 Faculté des Sciences et Ingénierie, Sorbonne Université, UFR 925 Physique, 75005 Paris, France; 3 Laboratoire Matière et Systèmes Complexes (MSC), Université Paris Diderot-Paris 7, UMR 7057 CNRS, 75013 Paris, France

**Keywords:** cardiolipin, mitochondria, cristae, curvature-based sorting, cone-shaped lipid asymmetry, nonbilayer structures

## Abstract

Mitochondria are known as the powerhouse of eukaryotic cells. Energy production occurs in specific dynamic membrane invaginations in the inner mitochondrial membrane called cristae. Although the integrity of these structures is recognized as a key point for proper mitochondrial function, less is known about the mechanisms at the origin of their plasticity and organization, and how they can influence mitochondria function. Here, we review the studies which question the role of lipid membrane composition based mainly on minimal model systems.

## 1. Introduction

Mitochondria are involved in different cellular functions, but they are essentially responsible for cellular ATP production through the so-called oxidative phosphorylation (OXPHOS). This process occurs in the inner mitochondrial membrane (IMM) in specific dynamic membrane invaginations called cristae. The shape of these structures and their number can differ depending on cellular type with different energetic requirements [1,2]. Cristae also undergo continuous cycles of membrane remodeling in physiological conditions [3]. If it is consensually accepted that their integrity is crucial for correct mitochondria functioning [4,5,6,7], less is known about the mechanisms by which membrane shape and composition operate to influence ATP production.

Different proteins have been evidenced to be involved in the morphological structuration of cristae. The most recognized of them are the MICOS complex, the protein OPA1 and the dimers of ATP synthase. Several reviews have summarized the knowledge on these proteins (see for example [5,6]), so it will not be the topic of the present review. Here, we are more interested in highlighting how the physicochemical properties of different mitochondrial phospholipids can trigger interesting behaviors susceptible to influence mitochondrial membrane morphology and organization. Indeed, the IMM contains almost 50% of cone-shaped lipids, which is unique in biological membranes [8]. These lipids have a geometrical shape promoting nonbilayer phases and bilayer curvature stress [9,10]. They are known to stabilize membrane proteins, but also to increase the capacity of the membrane to sustain fast remodeling required for biological functions such as fusion, fission, budding [11], and possibly in cristae-shape dynamics. This property, called morphological plasticity [12] is not only dependent on which lipid species are present, but also on how they are distributed. In fact, some lipids can be enriched in regions of high curvature and also asymmetrically distributed between the two leaflets of the bilayer. There also exists a lateral heterogeneity, caused either by the formation of proteo-lipid domains, or by lipid-driven mechanisms such as phase separation phenomena. The result can be the formation of localized domains in the plane of the membrane, which can have distinct compositions, structures, and biological functions. Finally, the structure of each individual lipid species and their interaction with others components of the membrane can change in response to physicochemical modifications of their surrounding environments such as pH, ionic strength and temperature, and can thus serve as a way to regulate or modify biological functions of the membrane in which they are situated in a non-specific way. Thus, the mitochondrial membrane, through its lipid composition, possesses a lipid membrane identity that can partly dictate its behavior [8].

An important part of the insight into membrane structure/function relationship in biology—and the concepts used to describe them—have come from studies of minimal model systems as models of biological membranes. These minimal model systems, bioinspired and based on a minimal number of key components identified for the respective function to analyse, provide a bottom-up approach to the study of biological membranes. They help to shape our intuition, to make quantitative predictions possible, and to guide experiments and the subsequent analysis and interpretation of the data. In this review, we looked at the recent studies performed using such in silico and in vitro minimal model systems which could explain for a part the dynamic behavior of cristae, and the consequences of their morphology on specific mitochondrial lipid sorting, lipid leaflet asymmetry and possible lateral heterogeneity. Finally, we discuss the possible functional implications of such organization and lipid composition.

## 2. Mitochondrial Cristae: Dynamics Bioenergetic Compartments

**Figure 1:**
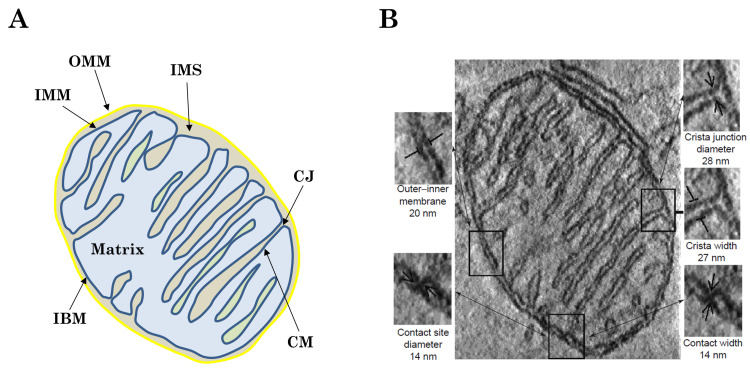
Structure of mitochondrial microcompartments. (**A**) Schematic representation of mitochondrial architecture. The outer mitochondrial membrane (OMM), inner mitochondrial membrane (IMM), inner boundary membrane (IBM), cristae junctions (CJ), intermembrane space (IMS), cristae membrane (CM) and mitochondrial matrix are indicated. (**B**) Electron micrograph from a cryo cut mitochondrion with antibody probing of OXPHOS complexes. The localization in the cristae membrane is obvious. Inset: detailed view of the two IMM compartments CM and IBM connected by the cristae junction (CJ). Scale bar: 150 nm. Reproduced with permission from Frey et al. [15], Trends Biochem. Sci.; published by Elsevier, 2000.

Mitochondria are complex organelles that arose from endosymbiosis over 1 billion years ago [13]. There are made up of two membranes. While the outer mitochondrial membrane (OMM) envelops the organelle, the IMM is subdivided into two compartments, the inner boundary membrane (IBM) and the cristae membrane (CM). The two membranes give rise to two aqueous compartments: the intermembrane space (IMS) and mitochondrial matrix (Figure 1). Cristae are multiple tubular or lamellar invaginations into the matrix separated from the rest of the IMM by cristae junctions (CJs). The CJs act as diffusion barriers and therefore contribute to delimiting cristae-bounded biochemical processes [14].

The IMM is among the protein-richest membrane of the cell [16]. In order to get a quantitative idea of such protein crowding, M. Schlame [17] describes a stoichiometric model of the IMM created on the basis of the molecular size and the concentration of the most abundant proteins and lipids suggesting that about half of the hydrophobic volume of the membrane is occupied by proteins and that the average surface-to-surface distance between proteins is <10 nm. Albeit this protein content is exceptional high, it still can be assumed that the IMM behaves as a semifluid arrangement of lipids and integrated proteins [18]. Importantly, cristae contain protein complexes responsible for the OXPHOS process: the oxidation of electron donors NADH and FADH${}_{2}$, produced by fatty acid oxidation and the citric acid cycle in the matrix, allow a flow of electrons through electron transport chain machinery which induced the pumping of protons from the matrix to the interior of the cristae. It allows the creation of an electrochemical gradient across the inner mitochondrial membrane which serves as the driving force (proton-motive force, PMF) for the production of ATP by the mitochondrial F${}_{1}$F${}_{O}$-ATP synthase according to the Mitchell’s theory [19].

The IMM morphology and organization are crucial for the assembly and proper function of the enzymes involved in OXPHOS process, as well as in the establishment of the proton gradient [4,5]. It was also shown that cristae shape and number can change depending on energy requirement [1,2,20] while, in different pathologies, alteration of mitochondrial function is most of the time associated with cristae destructuration [21,22,23]. This strongly suggests that a structure-function relationship exist at the cristae level.

### 2.1. Morphology and Structure-Function Relationship of Cristae

The presence of cristae was originally supposed to be the only consequence of the fold of the IMM due to the smaller outer membrane surface (baffle model [24]). However, results from electron tomography made this description more complex, since cristae are composed of different parts (Figure 1), with the presence of CJ, and the coexistence of tubular or lamellar structures [25]. Different proteins have been recognized as regulators of cristae structure (see reviews [5,26,27]). For example, the assembly of ATP synthase dimers into rows can induce membrane curvature and could be the first step in the formation of mitochondrial cristae [28]. Several theoretical models have also been developed to describe the IMM structure. It was first investigated how observed morphologies of restricted portions of the IMM can be used to infer thermodynamic information regarding typical membrane configurations [29,30]. Then, the model was completed in order to report the IMM morphological complexity (tubular and lamellar structure coexistence) [31]. Based on the minimization of the free energy of the system (IBM plus cristae), the stationary states found resemble the shapes of real cristae sufficiently closely that consistent and reasonable values of the thermodynamic properties—pressure difference across the IMM, membrane surface tension, tensile force required to stabilize the structure—of the cristae observed could be calculated. It was also speculated that protein such as OPA1 might apply this tensile force through a scaffolding mechanism [31].

**Figure 2:**
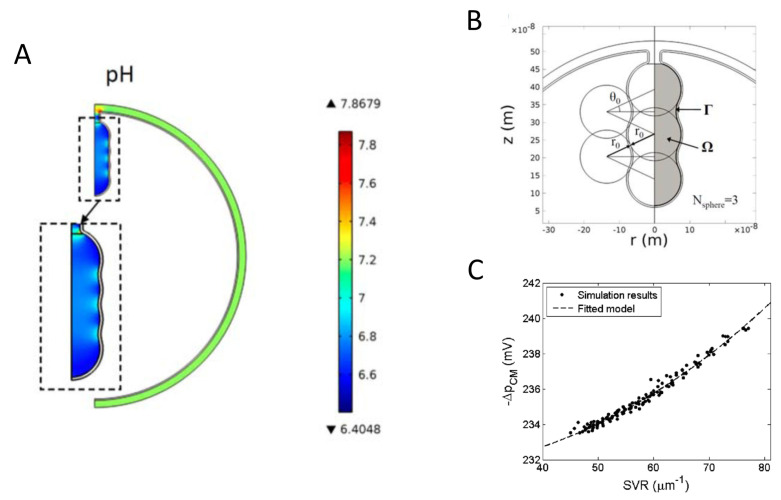
Mitochondrial model with a single cristae. (**A**) Proton concentration distribution (expressed as pH) in the inner mitochondrial space (cristae and non-cristae portions). (**B**) Geometry used to investigate the effect of detailed cristae morphology. (**C**) Effect of the surface-to-volume ratio (SVR) on the average PMF on the cristae membrane ($\Delta p_{CM}$). Reproduced with permission from Song et al. [34], Phys. Rev. E; published by the APS, 2013.

From a functional point of view, cristae structure was proposed to increase the inner membrane surface and thus to enhance the capacity of oxidative phosphorylation [26]. Cristae were further hypothesized to serve as a specialized compartment ensuring optimal conditions for ATP production by concentrating proteins involved in OXPHOS and reducing the mean distance between the different actors [32,33]. A local PMF is thus established more favorably in cristae than outside. This point was confirmed in a theoretical study [34] in which it was also showed that high proton concentration in cristae can be induced by the morphology-dependent electric potential along the outer side of the IMM (Figure 2A). It was also predicted that the cristae surface-to-volume ratio and surface area are more important than the cristae geometry for generating the PMF and determining the rates of ATP production (Figure 2B,C).

So, many hypothesis exist to explain the structure-function relationship existing in cristae, but an interesting axis of reflection is to understand how cristae shape can be modulated depending on energy state.

### 2.2. Plasticity of Cristae

Mitochondria are dynamic organelles of the cell whose shape constantly changes in vivo through fission and fusion events [35]. From a bioenergetic point of view, these mechanisms, associated with mitophagy, allow a quality control to keep optimal mitochondria function [36]. Other roles of such remodeling capacity have been also described in several physiological processes, including metabolic changes, redox signaling, calcium homeostasis and apoptosis [35,37]. In all cases, these dynamic events are usually associated with a rearrangement of the IMM morphology, especially at the level of mitochondrial cristae.

**Figure 3:**
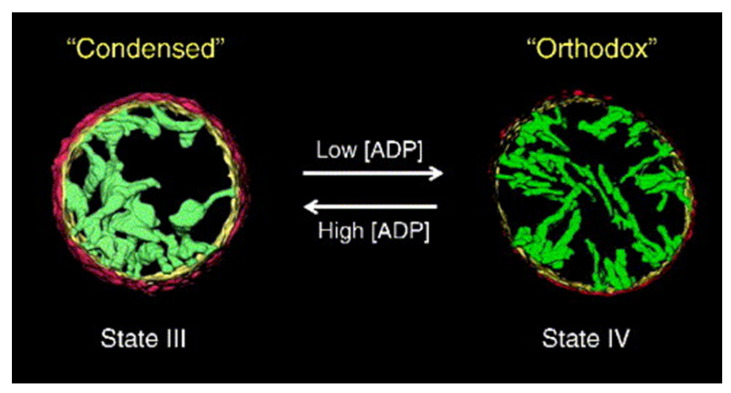
Illustration of the cross section of a mitochondrion observed under different metabolic conditions. The condensed morphology appears in the presence of high ADP concentrations, when mitochondria are producing ATP (state III), while the orthodox configuration occurs at low ADP concentrations, with no production of ATP (state IV). Reproduced with permission from Manella [25], Biochim. Biophys. Acta; published by Elsevier, 2006.

Intriguingly, it has been observated that the change of cristae structure depends on energetic state. In 1966, Hackenbrock [38] reported one of the earliest examples of mitochondrial dynamics when he noted that, in the presence of an excess of ADP (respiratory state III, active mitochondria), mitochondria showed a condensed conformation with large swollen intra-cristal space volume, while under ADP limiting conditions (respiratory state IV, rest condition), this volume was considerably decreased (orthodox conformation). This was the first evidence implying that cristae shape undergo dramatic changes in response to changes in metabolic state. These observations have been confirmed later by 3D electron tomography by the group of Mannella [25]. Both states are illustrated in Figure 3.

Different theories regarding how deformations of the mitochondrial membrane in general affect the ATP production rate have been proposed [3,7,34,39]. One of them was related to the elastic property of the IMM [39]. According to this theory, variations in the mechanical stress on the mitochondrial membrane can promote changes in inner mitochondrial membrane potential (assuming that the mitochondrial membrane behaves as a capacitor) and could serve as a feedback mechanism that controls the oxidative phosphorylation. Recent experiments [40] performed with purified ATP synthase reconstituted in liposomes also evidenced that the coupled proton pumping and rotation activities of the protein promote nonequilibrium membrane fluctuations at localized hot spots where the active proteins might be clustered. Thus, the activity of the F${}_{1}$F${}_{o}$-ATPase could favor the decrease in the bending stiffness of the membrane and the concomitant lowering of its surface tension, which could be the trigger for the change of the cristae morphology.

Today, more than 50 years after Hackenbrock’s observations, the study of mitochondrial membrane dynamics is a very active field of research. Cristae are now recognized as dynamic individual bioenergetic units [3]. This dynamic nature has been even directly observed using the new imaging techniques of super-resolution [41,42,43,44,45,46], capturing the continuous fusions/fissions occurring during time. However, to our knowledge, no clear relationship between cristae dynamics and mitochondria function has been demonstrated and visualized yet using such approaches. Some questions also remain about the molecular actors involved in this membrane plasticity and in mitochondrial function. They should be able to modulate membrane mechanical properties in a time dependent manner in response to an external trigger, and to change the OXPHOS efficency to modulate energy production. Despite a large amount of proteins potentially involved, IMM contains specific lipids which could support this role.

## 3. Lipids of Mitochondria: Focus on the IMM

Lipid compositions of both mitochondrial membranes exhibit features that set them apart from other cellular membranes. First, they contain mainly phospholipids, whereas only trace amounts of sterols and sphingolipids are present. Second, a hallmark of mitochondria is the high content of cardiolipin (CL), especially in the IMM [16,47]. The lipid composition of the IMM varies from that of the OMM. Phosphatidylcholine (PC) and phosphatidylethanolamine (PE) are the most abundant phospholipids in the IMM, comprising about 75% of total lipids. However, the concentration of PE is slightly higher in the IMM compared to the OMM, while the opposite is true for phosphatidylinositol (PI) [48]. One of the biggest differences between OMM and IMM lipid composition is the greater concentration of CL that is found in the IMM. Here, CL makes up about 15–20% of the total phospholipid mass [48]. Like all biological membranes, IMM lipid composition is tightly regulated and crucial for the organization and dynamics of cristae.

### 3.1. Presentation of the Main IMM Lipids

PC is the most abundant phospholipid of the IMM. It is a zwitterionic phospholipid, comprised of a hydrophilic choline head group bound to a glycerolphosphate molecule which contains two fatty acyl chains [49]. Bilayer-forming phospholipids like PC are cylindrically shaped with the fatty acid portions defining extended hydrophobic domains and the polar head groups defining the short hydrophilic domains along the length of the cylinder. The nearly equivalent diameters of the cylinder in both domains allow molecular packing that favors bilayers. PE is the second most abundant phospholipid in mitochondrial membranes. PE is also a zwitterionic phospholipid, composed of a glycerol phosphate backbone bound to two fatty acid chains in one end, and a polar ethanolamine head group in the other. The relative small-sized head group gives PE a conical shape, making it the most abundant nonbilayer forming phospholipid in mitochondria [49]. Finally, CL is a distinguishing component of the IMM and a unique phospholipid that not only differs from other membrane phospholipids regarding its membrane specificity, but also in its chemical structure. CL is an anionic molecule, and as opposed to other glycerophospholipids, it has a dimeric molecular structure which is composed of three glycerol groups, two phosphate moieties and four esterified fatty acyl chains, all bound to a compact polar head group [26,49]. From this structure results its distinctive conical shape. Under normal physiologic conditions, CL may only carry one negative charge at a time because the phosphates of CL are diastereotopically inequivalent, and thus ionize at two different pH levels [50,51]. This allows CL to possibly trap protons within its headgroup and thereby localize the proton pool near the surface of the IMM. The notion that CL contains two different pK${}_{a}$’s is still disputed and has been challenged and rejected by several groups [52,53,54] that claimed that the two ionizable phosphates are both deprotonated at physiological pH imparting a headgroup charge of −2.

The phospholipid diversity in the inner mitochondrial membrane is also influenced by variation in length and degree of unsaturation of fatty acyl chain present within each class of phospholipid. The role of the various fatty acyl chains of phospholipids is indeed crucial. For instance, the fluidity of a membrane at a given temperature or the extend of a lipid nonbilayer preference are determined by the acyl chain composition and nature [55,56]. The acyl chains of CL are highly tissue specific, varying from species to species. Most mammalian tissues have CL predominantly composed of 18 carbon unsaturated acyl chains, the vast majority of which are linoleic acid (18:2) [57]. In mammalian cardiac mitochondria for instance, this is the case for 85% of the total CL acyl chains, making tetralinoleoyl-cardiolipin [(18:2)${}_{4}$CL] the most common CL species of this tissue [58]. With the exception of the acyl chain remodeling of CL, the regulation of the acyl chain composition of mitochondrial lipids is still poorly understood [47].

### 3.2. IMM Lipid Shape Matters

**Figure 4:**
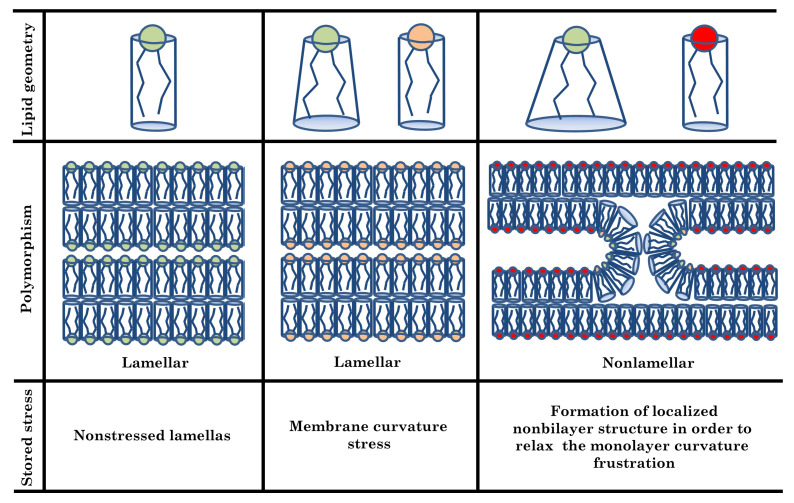
Molecular geometry of lipids and membrane stored stress. Monolayers made of cylindrical molecules of zero spontaneous curvature (SC) can form nonstressed lamellas (first column, green lipid). However, for nonzero SC, lipid molecules have to be reshaped to fit into a flat state, leading to membrane stress (second column, orange lipid). When the stress accumulated in the resulting bilayer is too big, the transition of the lamella into a nonlamellar phase is favorable. The transition begins when small interlamellar contacts having characteristic hourglass shape form (third column, red lipid), lipids with negative SC promotes formation of these localized nonbilayer structures [12].

**Figure 5:**
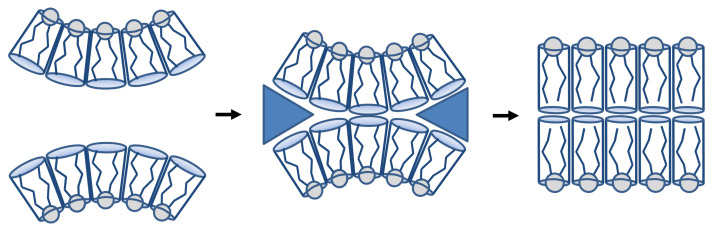
Curvature frustration illustrated for the case of a symmetric bilayer consisting of two monolayers that have an inherent desire to bend. In order to avoid energetically unfavourable voids (blue triangles), the two monolayers must lie flat back-to-back, resulting in a stored curvature elastic stress [10].

Not all phospholipid species, when hydrated, assemble in bilayers. There are many examples of lipids that have a preference for assembling as nonbilayer phases [59]. These differential nonlamellar-forming tendencies have long been explained with the relaxed molecular shape of a phospholipid [12]. Optimally, a phospholipid self-assembles into a supramolecular structure that reflects the relaxed shape of its molecule under given conditions (Figure 4). The essence of this shape concept can be captured and quantified with monolayer spontaneous curvature $C_{0}$ which describes the monolayer curvature of this supramolecular structure. In general, lipids with molecular shapes different from cylinders will form monolayers that either curve away or towards the polar/apolar interface ($C_{0}\neq 0$) [60] (Figure 5). For example, lipids with a negative spontaneous curvature ($C_{0}<0$) are prone to form non-planar structure like inverted hexagonal phases H${}_{II}$. When the phospholipids with different $C_{0}s$ are mixed, the resulting structure would exhibit a collective $C_{0}$ weighted by the relative amounts of the composing phospholipids [61].

In planar membranes, non-zero $C_{0}$ monolayers are forced into a flat topology, where they lie back-to-back— in order to avoid energetically unfavorable voids–leading to significant curvature elastic stress that is stored within the membrane [10] (Figure 5). However, this effect can only be tolerated up to a certain point. Upon further stressing the bilayer, a transition to a nonlamellar phase would be induced. Interestingly, the IMM contains a particularly high level of these cone-shaped lipids such as PE and CL (almost 50%). They assume a patent conical shape owing to the smaller cross-sectional area of their headgroups relative to their often unsaturated and kinked acyl chains. Whereas zwitterionic PE tends to form nonbilayer phases on their own ($C_{0}<0$) [59], anionic CL, owing to the electrostatic repulsion between its charged headgroup, necessitate charge neutralization by divalent cations, by changes in pH or by ionic strength to facilitate this phase transition (i.e., that $C_{0}$ varies with the environmental conditions) [54,62]. Interestingly, Chen et al. [63] have shown that the $C_{0,DOPE}$ has a clear dependence on temperature whereas it demonstrates a weak response to the [$Ca^{2+}$] variations at all the studied temperatures. On the contrary, $C_{0,[18:1]CL}$ is essentially independent of temperature at nearly each the [$Ca^{2+}$] examined but shows a response to the [$Ca^{2+}$] variation far more pronounced than for DOPE.

Due to the properties of its lipid constituents, the IMM could be under elastic stress and prone to the formation of nonlamellar local structure with possible functional consequences (see Section 5.3.2).

## 4. Physicochemical Properties of Only-IMM Lipid Systems

The main IMM lipids, and in particular CL, are naturally the basic building blocks of any IMM-mimicking minimal system. Therefore, an important amount of work has been aimed at understanding different CL-dependent properties of only-lipid systems. Numerous studies have probed the biophysical properties of binary mixtures of CL with PC or PE lipids whereas those investigate the properties of CL in mixed PC-PE lipid membranes are less frequent.

### 4.1. Interactions of CL with Other Phospholipids

The behavior of lipid monolayers composed of binary mixtures of bovine cardiac CL and egg PC (EPC) at the air/water interface of a Langmuir trough has been examined [64]. This study indicates that CL and EPC are fully miscible with each other at all proportions tested and that CL enhances lateral interactions between lipids within monolayer leaflets. Very similar results were presented in other monolayer film studies of mixtures of CL with respectively POPC or POPE in the Domenech’s work [65] and DPPC or DPPE in Sennato’s work [66]. It was also observed in both studies that the CL-PE systems—supported planar bilayers in [65] and supported monolayers in [66]—formed different lipid domains believed to be caused by the tendency of both lipids to form hexagonal phases. In silico attempts have also been made to model the effect of CL on different matrices (PC, PE, and mixed PC–PE for reference [67]) using both coarse-grained [68] and molecular dynamics simulations [67,69]. These studies suggest as well that the incorporation of CL into PC bilayers should have a significant ordering effect, predictions supported by the experimental observations of Khalifat et al. [70]. On the other side, Róg et al. [67] showed that the effects of CL in ternary membrane systems are complex and cannot be easily deduced from the corresponding ones in binary membranes. Indeed, the ternary mixture of PC, PE, and 10 mol% CL they modeled was only mildly condensed as compared to the corresponding CL-free binary PC-PE bilayer. Similarly, the results from the simulations by Pöyry et al. [71] and Wilson et al. [72] indicated a small condensation in a ternary lipid bilayer. Additionally, fluorescence experiments performed by Khalifat et al. [70] suggested that although the addition of 10% CL to PC bilayers (at pH 7.4) leads to condensation, there was no discernible effect on mixed PC-PE bilayers. Taken together, these studies suggested that CL induces a mild condensing or ordering effect in mixed PC-PE lipid bilayers as long as the concentration of CL remains less than or equal to the CL physiologically relevant composition (∼20 mol%).

### 4.2. Mechanical Properties of Only-IMM Lipid Systems

Because lipid bilayers can bend and stretch in ways similar to thin elastic sheets, physical models of bilayer deformation have utilized mechanical constants such as the moduli for bending rigidity ($\kappa_{c}$) and area compressibility ($K_{A}$) [73]. Indeed, in order to deform a lipid bilayer, the primary energetic cost comes from the bending and stretching of lipids. These modes of deformation alter the separation between polar lipid headgroups and regulate the amount of exposure of nonpolar lipid hydrocarbon tails to the aqueous medium, which also changes the energetic state of the lipids.

There are only few experimental studies on the mechanical properties of bilayers CL. In one of them, Nichols-Smith et al. [64] have shown that CL-containing bilayers are prone to create folds and adopt highly curved structures because both apparent area compressibility modulus and lysis tension decrease with increasing CL content in SOPC bilayers. These findings indicate that a lower stress is required to achieve a given change in membrane area and suggest a weakening of the cohesive strength of the membrane. This latter point has been confirmed in a study by Unsay et al. [74]. In these experiments, an increasing force is exerted on the supported lipid bilayer via an AFM tip until reaching the necessary force to pierce the membrane. CL decreased the breakthrough force of the bilayer in a concentration-dependent manner suggesting again that CL decreases the mechanical stability of the lipid bilayer. This result is assumed to be related to the CL propensity to form lipid arrangements beyond the canonical lipid bilayer [74].

Bilayer deformability is crucial for maintaining the highly curved cristae membrane. A more rigid bilayer accrues a larger energetic penalty when forced into curved morphologies. Membrane bending energetics depend on both the bending modulus $\kappa_{c}$ and spontaneous curvature of the lipid bilayer constituents [10], so both aspects may play a role in IMM morphology. The bending modulus is a mechanical macroscopic constant that describes the tendency of a certain material to oppose bending. Experimental measurements of pure CL [75] or mixed PC-CL bilayers [76] yield a bending modulus which is larger than that observed in pure PC bilayers indicating that CL stiffens the membrane. In the case of the IMM mechanics, this result has to be taken with caution because saturated tetramyristoyl [(14:0)${}_{4}$] CL and monounsaturated tetraoleoyl [(18:1)${}_{4}$] CL respectively used in these two studies differ from tetralinoleoyl [(18:2)${}_{4}$] CL, the most common CL species [58]. Indeed, even if to the best of our knowledge there is no measurement of $\kappa_{c}$ for tetralinoleoyl [(18:2)${}_{4}$] CL-containing bilayer, bending rigidity $\kappa_{c}$ values are known to be lower for polyunsaturated lipid-containing membranes [77,78]. Anyway, a reduction of the bending energy through the intrinsic curvature of the CL may be possible and related to spontaneous curvature of the CL-enriched cristae [79,80].

### 4.3. Non-Specific Regulatory Roles of IMM Lipids

Non-specific ways by which lipids fulfill their regulatory role are realized through the changes in membrane physical parameters, such as membrane hydrocarbon thickness, surface charge density, lipid head-group hydration, etc. Thus, understanding how physicochemical properties of lipids affect membrane properties is crucial in order to appreciate the importance of the phospholipid molecular species profile for maintaining membrane function in vivo. The particular IMM lipid composition—and the option to modulate its properties, for example by adding calcium [63,81,82,83,84] or lowering pH [54,70,85]—may therefore provide some important biomembrane regulatory mechanisms by giving the possibility to fine-tune the membrane properties. Some of these mechanisms will be developed in the next section.

## 5. The Intricate Link between Lipid Composition and Cristae Organization Revealed by Minimal Model Systems

In this Section, important results regarding membrane regulatory mechanisms based on only-lipid systems mimicking the IMM are highlighted. In Section 5.1, the role of CL on the formation and maintenance of dynamic tubular membrane invaginations is investigated while the cone-shaped lipid sorting within the IMM is presented in Section 5.2. Then, in Section 5.3, lateral heterogeneities within the IMM are described with a particular attention paid to the formation of nonbilayer structures (Section 5.3.2).

### 5.1. Role of CL in Cristae Biogenesis, Morphology and Dynamics

**Figure 6:**
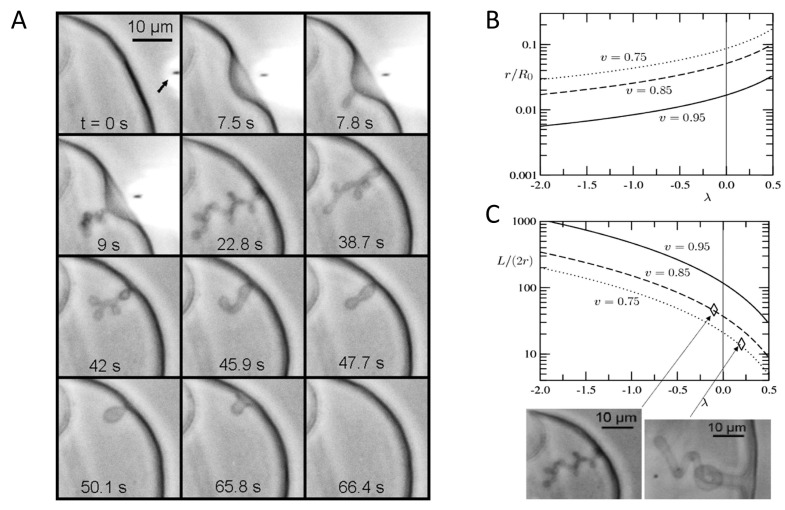
Cristae-like membrane invaginations. (**A**) Modulation of local pH gradient at membrane level of a cardiolipin containing vesicle induces dynamic cristae-like membrane invaginations. GUV is made of PC/PE/CL 60:30:10 mol/mol in buffer at pH 8. The local delivery of HCl (100 mM pH 1.6), which lowers the local pH, is carried out by a micropipette (its position is pointed by the arrow in frame t = 0 s).The induced membrane invagination (frame 22.8 s) is completely reversible (frames 38.7–66.4) as far as the acid delivery is stopped. Geometrical features of the tubular invagination calculated from the model as a function of the area reduction factor λ that is controlled by the acid delivery (the lower λ, the stronger acid effect): (**B**) Radius of the tube normalized by the radius of the initial vesicle. (**C**) Aspect ratio of the tube. Both are given for three experimentally relevant values of the reduced volume v of the initial vesicle. Two experimental illustrations are given for ν = 0.85 and 0.75 and L/(2r) ratios ∼40 and 14, respectively. Reproduced with permission from Khalifat et al. [86], Biophys. J.; published by Elsevier, 2008.

In 2008, Khalifat and coauthors [86] were the first to propose that phospholipid components, and especially CL, could be involved in the dynamics of cristae. Using giant unilamellar vesicles (GUVs) containing a lipid composition mimicking the IMM one, they showed that, when a local proton flow was introduced to the membrane (mimicking the proton flow present during the OXPHOS), it triggered the formation of cristae-like invaginations (Figure 6). Replacing CL by phosphatidylserine (PS) or PI cannot reproduce the same cristae-like morphology as the CL-containing GUV, nor using phosphatidylglycerol (PG) which gives noticeably different behavior [87].

A simple geometric model was proposed to describe the experimental observations. One can see in Figure 6B,C the geometrical features of the tubular invagination calculated from the model as a function of the area reduction factor λ that is controlled by the acid delivery. In agreement with the experimental results, it was obtained long and thin tubes for strong effect of the acid and large and thin tubes for weak effect of the acid. It was find also that the tenser the vesicle the thinner and longer the tubes. Thus, these results suggested that, in addition to a role of different proteins in the structuration of cristae [5], CL could play an additional role, either by initiating cristae formation, or at least by participating to their dynamics.

In later studies, the same authors proposed a theoretical description of local membrane deformations attributable to local pH variations [70,86,88,89,90]. Briefly, the asymmetric pH-induced changes of protonation of the charged headgroups and the resulting changes of the electrostatic interactions between such headgroups promote asymmetric local modifications of the lipid packing. This affects both equilibrium lipid density and monolayer local spontaneous curvature, resulting in a local deformation of the membrane [90,91]. Intermonolayer friction also plays an important role in the relaxation dynamics of these deformations. An additional model simulating the morphologies of the IMM at the mitochondrial scale for given pH profiles was introduced by another group [92], confirming the possible role of CL in cristae formation, and suggesting that within cristae, a lower local pH value could exist [93], as well as a higher CL proportion than the other parts of the IMM.

**Figure 7:**
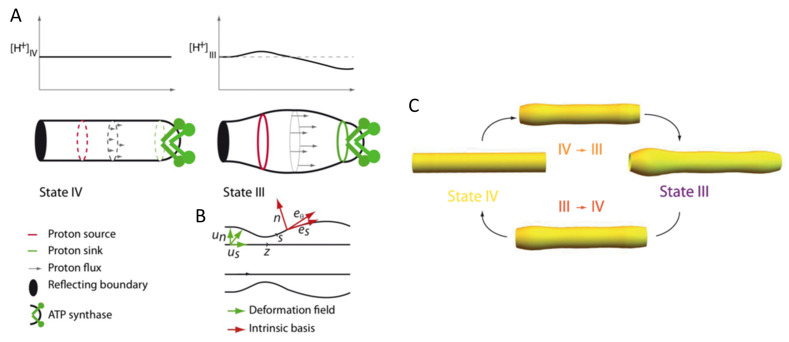
Mitochondrial cristae modeled as an out-of-equilibrium membrane driven by a proton field. Schematic representation of a cristae: (**A**) The plots represent the proton concentration on the surface in states III and IV. The tubes represent the shape of the invagination in state III and IV; (**B**) Deformation fields of the membrane and intrinsic basis of the deformed surface. (**C**) Changes in the cylindrical shaped model cristae morphology as the mitochondrion transitions between the two mitochondrial states IV and III. Reproduced with permission from Patil et al. [94], Phys. Rev. E; published by the APS, 2020.

Inspired by these results, Patil et al. [94] in a recent study modeled mitochondrial cristae using a pH-dependent Helfrich model. They showed that the shape of the cristae would oscillate in correspondence to an oscillating proton concentration field along the membrane (flowing from a source to a sink). The shapes of the two different mitochondrial states, as well as in the transition between the two, is shown in Figure 7. For a high proton concentration, the cristae-tube was deformed into a larger, more oval shaped tube (state III in Figure 7), whereas for no/low proton concentrations, the cristae-tube was purely cylindrical (state IV in Figure 7). This result is in agreement with Hackenbrock’s and Mannella’s observations of wide and bumpy cristae in the case of high ADP concentrations (and high production of ATP) which also imply a high proton flow along the IMM. In the opposite case however, there are low concentrations of ADP, i.e., no proton flux, and a more regular cylindrical cristae shape is observed.

Thus, these studies demonstrate that the pH-dependent behavior of a phospholipid such as CL is sufficient to mimic cristae plasticity as observed in mitochondria, the trigger being the proton flux occurring during the OXPHOS process.

### 5.2. Cone-Shaped Lipid Sorting within the IMM: Cristae Curvature and Leaflet Asymmetry

If cone-shaped lipids such as CL or PE are important for cristae function, it is important to know at which extent they are present in their vicinity. Biochemically, the measurement or the estimation of lipid content inside the cristae is tricky due to the difficulty to discriminate between cristae and the rest of the IMM, and to our knowledge, no protocol exists to isolate cristae fragment independently. Super-resolution imaging techniques will probably solve this problem, but right now, no experimental proof of a specific lipid composition inside mitochondrial cristae has been obtained. Still, such specificity can be anticipated for many reasons. Indeed, lipid composition of a membrane and of its leaflets is the result of several processes, including localized lipid metabolism, post-synthesis maturation, specific transport machinery and protein specific interaction [95]. In the latter case, several studies have shown that CL has a strong binding capacity for many proteins located within cristae, which can impact their structure, their function and stabilize them (see reviews [96,97]). For example, Cyt C is known to specifically interact with CL [98,99,100]. The ATP synthase, whose ultrastructure is depending on CL [101], interacts with CL via the F${}_{O}$ sector and the subunit DCCD-BPF [102]. The formation of supercomplexes also requires CL [103,104]. So the presence of CL inside cristae is a prerequisite for many mitochondrial protein functions. Considering that many of these CL-interacting proteins are located in the cristae part of the IMM, it is not unreasonable to think that cristae membranes might be enriched in CL, with possibly functional consequences as discussed later.

Specific lipid composition of cristae can also resulted from physical constraints linked to the intrinsic properties of the lipid. In particular, PE and CL possess a molecular relaxed shape which could drive specific localization induced by a spatial variation of curvature. Because cristae are nanostructures with a high curvature, it is tempting to speculate that such morphology will facilitate PE and CL sorting and asymmetry between both leaflets of the cristae membrane, and thus create specific lipid composition in cristae membrane.

#### 5.2.1. CL Enrichment Inside Cristae: The Role of Membrane Curvature

Numerous simulations have been performed to predict how cone- and inverted-cone-shaped lipids will behave in curved membranes [79,105,106,107]. For example, it was shown that an appreciable lipid sorting can occur when a very high curvature is induced by pulling a bilayer nanotube from a flat membrane [106]. In particular, CL is expected to localize preferentially in highly negative curved region [79,107]. For less curved membranes, only a weak curvature preference is obtained due to the small size of lipids [108,109] in contrary to proteins [110,111]. It was proposed that efficient lipid sorting could occurred if lipid-lipid interactions or lipid-protein interactions exist, in order to reach a sufficient size to sense curvature [112,113].

**Figure 8:**
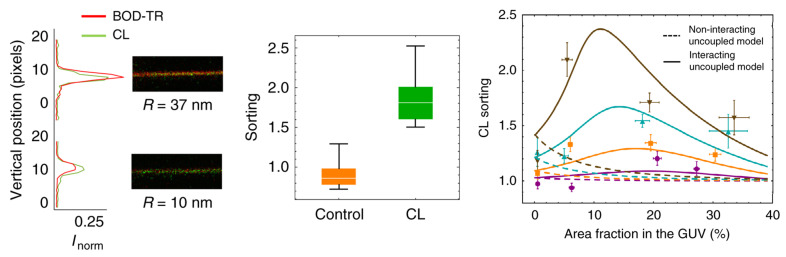
(**Left**) Images and intensity profiles of tubes pulled from GUVs containing CL for large (R ≈ 37 nm) and small (R ≈ 10 nm) tube radii. (**Middle**) Box plots comparing the sorting ratio for curved tubes pulled from GUVs containing green fluorescent lipids. CL is enriched in the tubes comparing with the lipid control. (**Right**) CL enrichment as a function of CL density in GUVs for four ranges of tube curvature. Dashed and solid lines represent respectively the minimum square fit to the non-interacting and interacting uncoupled model (no or possible CL-CL interactions). Reproduced with permission from Beltrán-Heredia et al. [80], Commun. Biol.; published by Nature Publishing Group, 2019.

Until now, no experimental proof has been provided showing a CL enrichment inside cristae. However, it is possible to test the hypothesis that membrane curvature can induce CL sorting using biomimetic membrane model. In a recent study [80], experiments were performed on GUV containing various amount of CL and EPC. Nanotubes of different diameters were pulled using an optical trap and a biotyl/streptavidin system, allowing different nanotube radius within the range of 8–40 nm. Using a specific fluorescent CL lipid, they were able to measure CL sorting as a function of membrane curvature and CL concentration (Figure 8). They shown that membrane curvature induce CL sorting in the GUV, and that the enrichment in the nanotubes were increased with an increasing curvature (not visible for nanotubes with a radius larger than 10 nm), and with increasing amounts of CL (up to a certain optimal value). In order to explain these results, they also suggests the existence of attractive CL-CL interactions. For the lipid sorting to occur, it must be energetically favorable, and generally there are two opposing factors involved: the bilayer relaxation due to negatively curved lipids in negatively curved membranes versus the decrease in mixing entropy obtained by sorting lipids by curvature. For a single lipid, the mixing entropy outweighs the bilayer relaxation, and the sorting would not occur. However, if one consider that there is an attractive force between the lipids, the sorting becomes favorable. Using a theoretical model accounting for CL-CL attractive interactions, they were able to reproduce their experimentally obtained values (Figure 8, Right).

These experiments shown that the membrane shape alone can modulate CL distribution in the membrane without any help of proteins. The only requirement is the presence of CL clusters containing at least ten CL molecules. If this is the case, considering the similarities between the nanotubes and mitochondrial cristae (both with respect to curvature and CL content), one could expect CL enrichment in cristae, but also the existence of CL enriched domains. Furthermore, the article also predicted that CL would preferably accumulate in the negatively curved leaflet, which lead to an asymmetric CL distribution in the membrane [80].

#### 5.2.2. CL Asymmetric Distribution between Cristae Leaflet: Really More CL on the Matrix Side?

The existence of CL asymmetry between both leaflets of inner mitochondrial membrane has been a matter of debate for a long time, and the question still remains due to contradictory results. In the 80–90’s, using mainly isolated mitochondria, mitoplasts or submitochondrial particles from mammalian cells, and biochemical approaches (immunological methods and phospholipase A2 digestion), different groups evidence a CL enrichment on the matrix side [114,115]. On the contrary, using spectrophotometry (Adriamycin binding) or fluorescence (CL dye NAO), other groups showed a small enrichment of CL on the IMS side [116,117]. Interestingly, in yeast, such distribution could depend of the level of oxidative phosphorylation and substrates availability [117,118]. It could imply that rapid translocation (on the orders of minutes) of CL could exist between leaflets [119]. However, concerns have been emitted concerning the dependence of CL labeling (NAO) in respect to the mitochondrial membrane potential [120,121].

Since these earliest 80–90’s studies, to our knowledge, no new study has been performed to assess CL distribution in IMM, and it is classically assumed that CL is mainly in the matrix leaflet side [16,48,122]. However, this conclusion relies mostly on the early results obtained in mitoplasts and submitochondrial particules (SMP) where cristae morphology is usually lost. So it is not known if in cristae, the same CL distribution exist. Cristae possess high curvature (negative in the IMS side, positive in the matrix side), and as explained before, CL should be preferentially located in negative curvature region, so in the monolayer leaflet facing the cristae lumen (IMS side) [26,80]. Indeed, it should confer stability to the curved cristae membrane. This could be also the case in contact sites where CL has been found in large amount, but in this case, the negatively curvature is on the matrix side. So, even if CL is synthesized on the matrix leaflet side, it could be rapidly recruit on the other leaflet, possibly by specific enzymes such as flippases or scramblases [123], or driven by a curvature-mediated mechanism [124].

**Figure 9:**
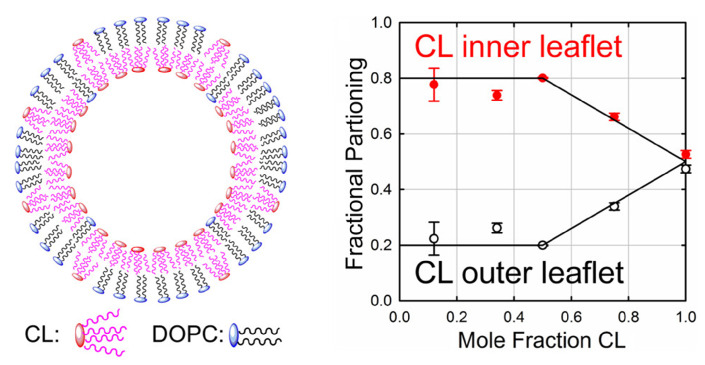
CL asymmetric distribution between the inner and outer leaflet of a LUV. The LUV have a diameter of 100 nm and are made of CL/PC with different CL concentrations. Reproduced with permission from Elmer-Dixon et al. [125], J. Phys. Chem. B; published by the ACS, 2019.

In line with this last assumption and the conclusions of Beltran-Heredia’s paper [80], a recent paper [125] studied CL distribution between the bilayer leaflets of large unilamellar liposomes (LUV, 100 nm diameter). They use the fluorophore TTAPE-Me (1,1,2,2-tetrakis[4-(2-trimethylammonioethoxy)-phenyl]ethene), which fluoresces upon binding to anionic head groups of lipids, but especially CL. The liposomes were made with a varying CL content ranging from 10 to 100% (Figure 9). By measuring the fluorescence associated to CL in the liposomes, they noticed that when they increased the mol fraction of CL in the CL/PC containing LUV, the fluorescence intensity did not increase accordingly. In fact, the fluorescence intensity was systematically lower than what was to be expected for an unbiased partitioning of CL in the inner and outer leaflet. For the mixed CL/PC vesicles with a CL fraction of 0.5 or less, the fractional partitioning of the outer leaflet was found to be 0.2. This result, which is illustrated in Figure 9, indicates that CL has a 4:1 preference for the negatively curved inner leaflet of the vesicle.

Replacing CL by PG, which is a cylindrical shaped anionic phospholipid, revealed that PG does not inhibit any preferential location with respect to the two leaflets, and it was concluded that the accumulation of CL in the inner leaflet is due to its conical shape only. Basing their arguments on previous studies, including the work of Beltràn-Heredia et al., Elmer-Dixon et al. also suggest that the asymmetric distribution of CL must be due to attractive lipid-lipid interaction as a way to compensate for the mixing entropy. They concluded that due to the tubular structure of mitochondrial cristae and narrow diameter of 30 nm, CL asymmetry could occur in cristae. So, there are many reasons to expect an enrichment of CL in the cristae lumen leaflet (IMS side), either due to the intrinsic nature of CL (cone shape), or due to the presence of favorable lipid-lipid interactions or protein-CL complexes. It implies that the physicochemical properties of the cristae membrane, especially in the IMS side could be more specific than we sought. In any cases, it also means that CL domains/clusters could exist in the cristae lumen leaflet, which can have again functional consequences. But, in intact cells, as explained before, experimental validations of this CL localization are required with new approaches that keep intact mitochondrial cristae.

#### 5.2.3. The PE Case

Only a few papers have focused on the role of PE in mitochondrial structure and function [126,127,128,129], and even less on a possible curvature-sensitive behavior. PE don’t carry negative charge in the physiological pH range, but possesses a conical shape like CL. One earlier modeling has shown, by considering a simple two-lipid model of the cristae (containing PC and PE), that lipids will redistribute themselves to reach a 7% difference between leaflets, since the resulting decrease in bending energy is smaller than the entropic penalty [30]. Another modeling also suggested that the presence of cone-shaped lipid such as PE could facilitate sharp bends at cristae junctions [29]. PE sorting due to membrane curvature has been also demonstrated experimentally using biomimetic membrane model [130], but two in silico studies predicted that PE, in contrary to CL, does not partition significantly [79,107].

### 5.3. Lateral Membrane Organization within Cristae-Like Membranes

Whereas there are numerous evidences highlighting the presence of lipid-driven domains in various organelles, only some clues of the existence of such domains are suggested in mitochondria [131]. *A priori*, there is no reason why the mitochondrial membranes—and in particular its cristae part— should not follow the general principle of membrane compartmentalization, especially since the existence of mesoscopic CL accumulation have been observed in bacteria [132,133,134]. Such clustering has been proposed to be driven by a curvature–mediated mechanism and can serve to recruit proteins to the poles [135,136]. According to the thermodynamic model developed then [124,137], clusters of CL are formed naturally due to the constraint of the membrane by the rigid cell wall, and these clusters are large enough to localize to the poles of the cell due to curvature. This model was also supported by experimental analysis of CL localization in *E. coli* cells with engineered shapes [138]. Interestingly, some conclusions of the thermodynamic model developed to describe the bacterial localization of the CL domain [137] seems relevant for cristae membrane lateral compartmentalization. For such a clustering to appear, the membrane concentration of CL has to exceed a critical value (below which entropy prevents the formation of domains large enough for stable polar localization) that is lower in curved geometries, one feature highly probable in CL-enriched highly curved cristae. Furthermore, the observed CL curvature-driven sorting described in Section 5.2.1 is shown to occur only if there is CL clustering [80]. So, it is particularly interesting to see if cone-shaped phospholipids, and in particular CL, form domain in cristae similar to CL-enriched domains observed in bacteria. To do so, a range of lipid model systems, involving mono- or bilayer of different morphologies and compositions can conveniently be used to investigate lateral membrane structures.

#### 5.3.1. Is Lateral Compartmentalization Detected in Only-Lipid Systems?

**Figure 10:**
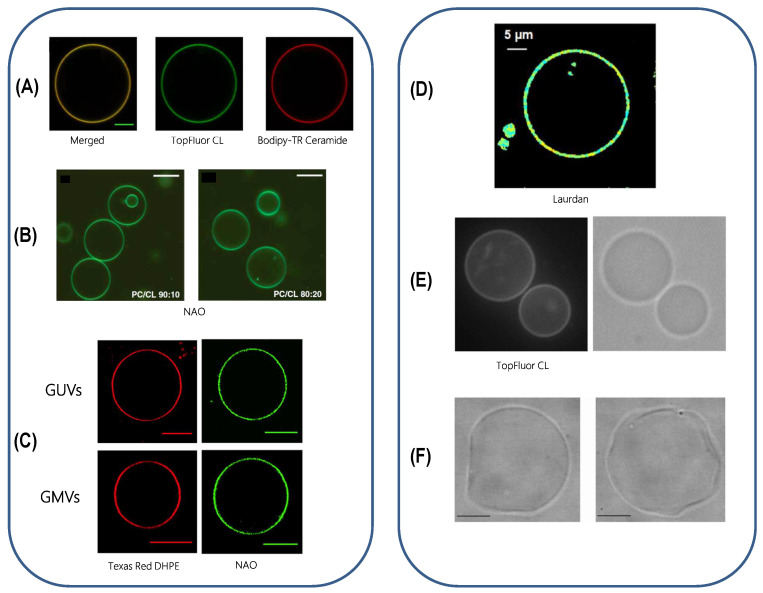
Giant vesicles as model systems. (**A**) Confocal image of a GUV made of EPC/CL 60:40 mol% showing the distribution of Top-Fluor CL (green channel) and Bodipy-TR Ceramide (red channel), 22 ${}^{\circ }$C, scale bar is 5 µm, [80]. (**B**) Confocal microscopy of GUVs made of PC/CL 90:10 and 80:20 mol%, room temperature, scale bar is 50 µM, [139]. (**C**) GUVs made of composed of (18:0–22:6)PC/(16:0–20:4)PE/(18:2)${}_{4}$CL/DOPI/DOPS/Chol (39.9:30:20:5:3:2 mol% and giant mitochondrial vesicles (GMVs) from native phospholipids extracted visualized by Texas Red DHPE (left) and by NAO (right) at 0.1 mol%. room temperature, scale bars are 10 µm, [100]. (**D**) Laurdan GP image of isolated EPC/EPE/CL (50:25:25) mol% membrane, room temperature, scale bar is 5 µm, [141]. (**E**) Fluorescence and optic microscopy images of POPC/DOPE/CL (49:30:20) GUV containing 0.5 mol% TopFluor-CL, 25 ${}^{\circ }$C, [140]. (**F**) Phase contrast images of GUVs made of POPC/CL (70:30) mol%, 23 ${}^{\circ }$C, [142]. Reproduced with permissions from respectively Beltrán-Heredia et al., Pennington et al., Jalmar et al., Kawai et al., Cheniour et al., and Tomšié et al. [80,100,139,140,141,142], in respectively Commun. Biol., J. Biol. Chem., Cell Death Dis., J. Phys. Chem. B, Biochim. Biophys. Acta, and J. Chem. Inf. Model; published respectively by Nature Publishing Group (2019), Elsevier (2018), Nature Publishing Group (2010), ACS (2014), Elsevier (2017), and ACS (2005).

Relatively few studies regarding the possible compartmentalization of inner membrane mimicking only-lipid systems have been performed. In some of these, experimental strategies based on epi-, confocal and two photon excitation fluorescence microscopy techniques have been employed to study the lateral structure of membranes using giant vesicles as model systems [80,100,139,140,141].The most common approach relies in the partition properties of particular fluorescent probes into the possible coexisting lipid domains (Figure 10: for experiments A, B, C and E) where the fluorescence intensity images obtained will be limited to information about the lipid domain’s shape and size. In the case of Laurdan labeled membrane (Figure 10: experiment D), local physical properties of the possible lipid domains were used to associate their presence with equilibrium thermodynamic phases. In the experiments presented in Figure 10E,F, phase contrast imaging allows to follow the GUV morphology [140,142]. As it can be seen in the left panel of Figure 10, for some IMM-mimicking lipid compositions, the fluorescent probe used is homogeneously distributed throughout the membrane, and no mesoscopic domains are observed. On the contrary, for others IMM-mimicking lipid compositions, heterogeneous membranes are evidenced either by Laurdan generalized polarization (GP) imaging, by Topfluor-CL clustering, or by local curvature changes (respectively Figure 10D–F). It should be noted that fluorescence microscopy only detects mesoscopic lipid domains (larger than 1 m). So, the existence of nanosized domains cannot be ruled out for the lipid compositions studied in the experiments presented in the left panel of Figure 10. Nanoimaging with atomic force microscope (AFM) could therefore be useful and the formation of laterally segregated domains has in fact been reported with AFM for binary mixtures of CL and PE lipids [65,66] and in a ternary mixture of PC, PE, and CL [143].

Anyway, an unambiguous conclusion of a possible lipid-driven lateral heterogeneity mechanism in IMM-mimicking systems is still difficult to make because of the disparity of the described experiments led with different lipid compositions, different lipid molar proportions, and differences between the length and degree of unsaturation of the hydrocarbon chains of the CL and non-CL components of the mixtures.

However, recent technical advances in nanoscopy should provide definitive evidences for the existence of nanosized domains in precisely designed IMM-mimicking systems, possibly in highly curved tubes that would well mimic the cristae membrane.

#### 5.3.2. Possible Lamellar/Nonlamellar Phase Coexistence

As seen before, the negative spontaneous curvature of PE and CL could lead to bilayer-disrupting properties. In particular, the ability of CL to organize into nonlamellar phase suggests that CL might promote specific localized structures within the IMM. To further investigate this possible lamellar/nonlamellar phase coexistence of CL-containing membranes, a comprehensive ${}^{1}$H and ${}^{31}$P nuclear magnetic resonance (NMR) analysis was recently performed by Lopes et al. [144] in order to define the structure, lipid phase state and thermotropic behavior of lipidic systems with respect to their phospholipid composition, in particular their CL content. Binary and ternary mixtures of POPC, POPE, and a natural extract of CL (CL${}_{mix}$) were used to focus on models being in a close proximity to the natural IMM.

**Figure 11:**
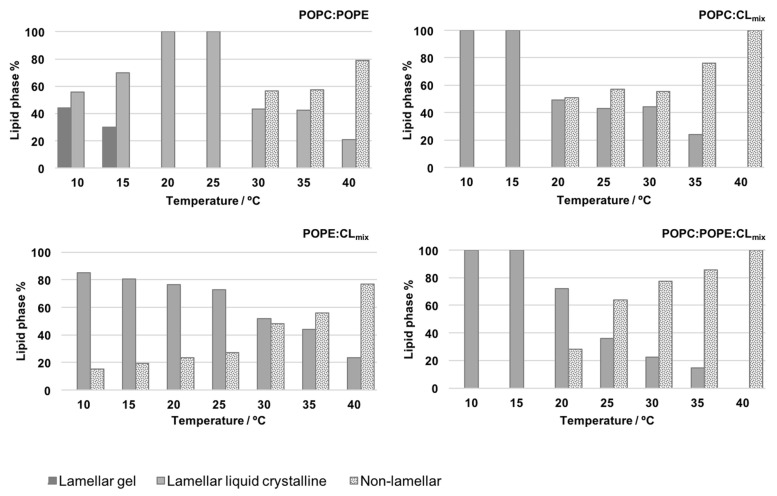
Relative quantitative distribution of different lipid phases as function temperature of POPC/POPE (0.5:0.5), POPC/CLmix (0.7:0.3), POPE/CLmix (0.6:0.4) and POPC/POPE/CLmix (0.5:0.3:0.2) in the studied temperature range. pH 7.4, 0.1 mM NaCl. Reproduced with permission from Lopes et al. [144], Biochim. Biophys. Acta; published by Elsevier, 2017.

${}^{1}$H and ${}^{31}$P NMR results revealed that CL affects the conformation, mobility and structural order of the phospholipid molecules. According to ${}^{1}$H NMR results, CL disturbs the overall structure and packing order of membrane demonstrated with the decrease of the line broadening and shift of all resonances. The ${}^{31}$P NMR line shape analysis confirmed that, at distinct temperatures, different lipid phases coexist in the systems, and their type and quantitative distribution are CL dependent (Figure 11). In particular, the results indicate that at physiological temperature the lipids in PC/CL and PC/PE/CL are predominantly in nonlamellar phase state. Interestingly, by using intact mitochondria and model membranes mimicking the IMM, Gasanov et al. [102] observed as well that a raise of temperature (8 to 37 ${}^{\circ }$C) elevated the formation of nonbilayer structures. Their data support also a model by which H${}^{+}$ and CL interplay to facilitate the formation of such structures, a result also obtained by Kooijman et al. [54].

Anyway, CL may play an important role in the lateral organization of IMM models. It seems to have the ability to form clusters or localized nonbilayer structures that are able to alter membrane physical properties in a local-manner and to recruit specific membrane proteins. For example, in their paper’s conclusions, Gasanov et al. claimed that these CL-mediated nonlamellar structures may favor the formation of specialized domains that serve to cluster H${}^{+}$ and ATP synthase complexes as a mechanism to enhance H${}^{+}$ translocation to the F${}_{O}$ sector (the functional facet of these domains will be developed in Section 6.3). Like rafts in plasma membranes, such membrane lateral compartmentalization, with a specific lipid and protein composition, might provide the possibility to dynamically modulate the activity of only a limited group of the IMM proteins. Such mechanism support the conclusion made in the paper of Jouhet [145]: “This might comfort the theory that cells adjust their membrane lipid composition in response to perturbations in order to maintain bilayer stability, but keeping the bilayer close to a point of instability, where a confined transformation to some nonbilayer structure would tend to occur”.

## 6. Discussion: Lessons from Minimal Models for Lipid Functional Implications

Alterations of lipid synthesis, integrity or post-maturation can have functional consequences in vivo. In the case of mitochondria, the best example is the Barth syndrome where mutations in the tafazzin gene induce CL remodeling alteration and lower CL abundance [146]. In this pathology, cristae organization is disturbed, associated with a impairment of the OXPHOS functionality [21,147], underlying the possible interconnection between morphology, function and lipid composition at the level of cristae. PE in vivo also seems to play an important role in mitochondrial structure and function [126,127,128], and can compensate CL deficiency [148]. Thus, the cone-shaped characteristics of these lipids and their physicochemical properties made them important molecular players involved in the structure-function relationship existing in cristae as well as in the functioning of OXPHOS process.

As explained in Section 2, an important characteristic of mitochondrial cristae structure is to optimize energy production by creating a local confined environment possessing specific mechanical properties. The experiments using minimal models described above predict that cristae lipid composition could be very specific for physicochemical reasons. Thus, lipids such as CL could contribute to some functional properties of cristae.

### 6.1. Cristae Plasticity: CL as a Sensor of Proton and Calcium Concentrations for the Modulation of Membrane Properties

The presence of proton-sensitive lipids like CL could allow the modulation of cristae morphology in parallel to energy production [94]. Indeed, during the OXPHOS process, protons, which are pumped from the matrix to the interior of cristae, can interact with CL and modify mechanical properties of the membrane, which is necessary to support energy production [39]. In the same manner, calcium ion can also change CL properties when present at a certain extent [83]. Biologically, both situations are relevant: for example, in muscle cell, in addition to the proton flux, calcium uptake occurs in mitochondria, possibly in a beat-to-beat fashion, which can enhance the activities of different OXPHOS proteins [149]. Calcium interaction with CL could be another way to boost respiration by modifying mechanical membrane properties of cristae, and thus the efficiency of ATP production. So CL is a good candidate to sense variations in energy demand via its ability to interact with protons and calcium.

Additionally, the increase of cristae surface-to-volume ratio could be also facilitate by cone-shaped lipids, since they can facilitate membrane fusion between adjacent cristae [150]. Such events are necessaries to elevate mitochondria capacity to produce ATP at times of increased metabolic demand or decreased fuel supply [104], and can also explain the condensed-to-orthodox transition observed by Hackenbrock [38]. So again, the physicochemical properties of CL and its ability to sense proton and calcium levels can participate to the plasticity of cristae, which is a prerequisite to adapt energy production to energy demand.

### 6.2. Impact of CL Enrichment in OXPHOS Functioning: To Facilitate Proton Circuit along the Membrane

**Figure 12:**
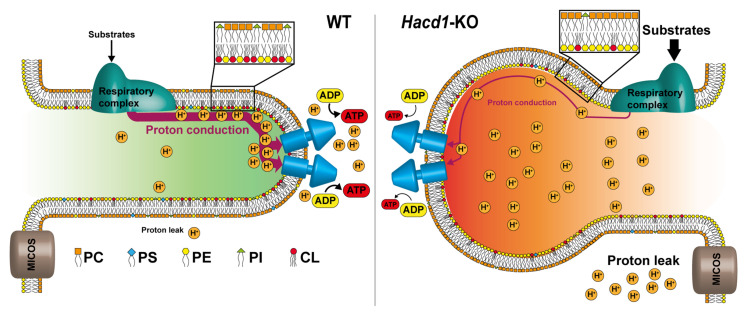
A proposed model of the molecular mechanism underlying the mitochondrial uncoupling in HACD1-deficient muscles. In wild type conditions, anionic lipids included in the cristae lumen leaflet of the IMM contribute to the translocation of protons to the tip of the cristae, where ATP synthase oligomers concentrate. In *Hacd1*-KO mice, the decreased content of anionic lipids changes cristae shape, reduces efficiency of proton translocation, hence impairing ATP production.

The presence of CL at a higher extent in the cristae lumen leaflet as demonstrated by in vitro systems and predicted by in silico studies, can directly affect the functioning of the OXPHOS process independently on their effect on OXPHOS protein activity. In a recent study, Prola et al. [23] showed that in a CL-deficient mice induced by the mutation of the HACD1 gene, cristae shape was altered, and a reduced coupling efficiency in the respiratory chain (diminution of the yield of ATP produced by oxygen consumed) was observed, with no change in respiratory chain complex activity and in proton gradient formation. The addition of CL to isolated mitochondria from *Hacd1*-KO mice (by fusing with CL liposomes) rescued their coupling efficiency, pointed out the specific role of CL content in the efficiency of OXPHOS process. The authors proposed a possible role of CL in facilitating the lateral transfer of protons from respiratory complexes to ATP synthase (see Figure 12). This hypothesis relies on earlier studies proposing an alternative mechanism to the classical Mitchell’s theory, based on local proton circuit on the membrane [151,152,153].

Indeed, a proton transfer from a proton generator to an acceptor is faster if occurring on the membrane rather than having the protons released into the aqueous bulk. Thus, a localized coupling can occur very rapidly on the membrane as opposed to the delocalized coupling [154,155]. Different lipids can participate to this proton transport circuit at the membrane’s surface, but CL, due to its ability to “trap” protons [156] and its asymmetric enrichment within the leaflets of cristae, is the best candidate, and could facilitate such proton transport and optimize mitochondrial function.

### 6.3. Role of Lipid Lateral Heterogeneity in ATP Synthase Functioning

**Figure 13:**
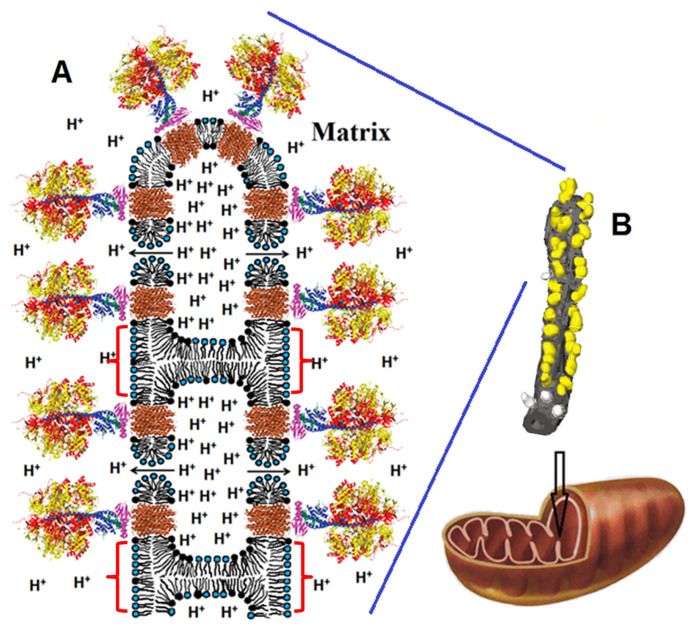
Illustration of Gasanov et al’s hypothesis suggesting the inverted micellar organization of cristae membranes (shown by red brackets). Reproduced with permission from Gasanov et al. [102], Biochim. Biophys. Acta.; published by Elsevier, 2018.

Cone-shaped lipid such as CL can also participate in specific membrane organization which will impact cristae function. Gasanov et al. [102] hypothesize that CL, the ATP synthase, and a proton gradient facilitate the formation of nonbilayer structures in the IMM which in turn stimulates ATP synthesis. They suggest that the low pH arising from the proton gradient makes CL transition from a bilayer to a nonlamellar phase, which leads to membrane sections organized as inverted micelles. These sections are predominantly composed of CL, which will localize to the highly curved sections of the micelle. The resulting membrane domains attract the F${}_{0}$ part of the ATP synthase and promotes the clustering of protons. The enriched CL concentrations increases the membrane’s ability to hold on the high proton concentration as the negatively charged headgroup of CL serve as “proton traps” which accelerate proton translocation and subsequent ATP synthesis. As shown in Figure 13, the inverted micellar organization of cristae decreases the cristae volume in which the proton accumulates, effectively increasing the proton concentration. If the proton gradient becomes too high, Gasanov et al. suggests the formation of short-lived toroidal-like pores as a way of rapidly decreasing in the transmembrane potential by releasing protons into the mitochondrial matrix. In conclusion, they attribute CL a role as a modulator of ATP synthesis via its properties to locally self-organize in nonbilayer structures which impact OXPHOS functioning.

So, again, cone-shaped lipid such as CL can participate actively to the optimal function of OXPHOS process, in a way that can be understood and analysed using minimal models, taking into account, of course, the results obtained in intact mitochondria.

## 7. Conclusions

In this review, we tried to provide a comprehensive overview on the knowledge of the mitochondrial lipid properties which could help to decipher the functioning of mitochondrial cristae. We focused on the results obtained using minimal model systems because they allow the study of specific lipid properties more directly than in biological systems. These approaches suggest that the presence of a high content of cone-shaped lipids like CL in curved membrane such as cristae could be an important feature of ATP production regulation. Of course, the existence of CL enrichment in cristae needs to be demonstrated in intact cells, perhaps using super-resolution imaging techniques. The dynamic structure-function relationship existing in cristae also needs confirmation in a more straightforward way. Finally, experiments in minimal models mostly utilize synthetic lipids that don’t take into account the great diversity of lipids existing in biological membranes. Using more systematically natural lipid extracts would be more relevant in future experiments.

The goal of this review is not to minimize the role of many proteins probably involved in specific tasks, but more to encourage readers to consider lipid properties as an important source of possible biological functions. For historical and technical reasons, most therapeutic strategies target proteins, whilst lipids are often neglected. However, the 40,000 identified lipids (www.lipidmaps.org, accessed on June 2020) massively contribute to the architecture and functions of the cell; they represent an equivalent amount of bioactive molecules that, to a large extent, determine the functioning of membrane proteins. Modulating their levels, localization or metabolism will obviously become an emergent, alternative therapeutical issue. Better understanding the role of specific mitochondrial lipids, especially using minimal model systems, in the dynamics of cristae, and in the functioning of the OXPHOS process, can open mechanistic modulating opportunities, which feasibility can be assessed in cellular models.

## Data Availability

Not applicable.

## Abbreviations

The following abbreviations are used in this manuscript:

ATP	Adenosine triphosphate
OXPHOS	Oxidative phosphorylation
IMM	Inner mitochondrial membrane
MICOS	Mitochondrial contact site and cristae organizing System
OPA1	GTPase optic atrophy 1
OMM	Outer mitochondrial membrane
IBM	Inner boundary membrane
CM	Cristae membrane
IMS	Inner mitochondrial space
CJs	Cristae junctions
NADH	Nicotinamide adenine dinucleotide hydrogen
FADH_2	Flavin adenine dinucleotide hydroquinone form
PMF	Proton-motive force
F${}_{1}$F${}_{o}$-ATP synthase	F-type ATP synthase
CL	Cardiolipin
PC	Phosphatidylcholine
PE	Phosphatidylethanolamine
PI	Phosphatidylinositol
DOPE	1,2-dioleoyl-sn-glycero-3-phosphoethanolamine
EPC	Egg phosphatidylcholine
POPC	1-palmitoyl-2-oleoyl-glycero-3-phosphocholine
POPE	1-palmitoyl-2-oleoyl-sn-glycero-3-phosphoethanolamine
DPPC	1,2-dipalmitoyl-sn-glycero-3-phosphocholine
DPPE	1,2-dipalmitoyl-sn-glycero-3-phosphoethanolamine
SOPC	1-stearoyl-2-oleoyl-sn-glycero-3-phosphocholine
GUV	Giant unilamellar vesicle
PS	Phosphatidylserine
PG	Phosphatidylglycerol
Cyt c	Cytochrome c
DCCD-BPF	Dicyclohexylcarbodiimide-binding protein of the $F_{0}$ sector
NAO	Nonyl acridine orange
SMP	Submitochondrial particle
LUV	Large unilamellar vesicle
TTAPE-Me	1,1,2,2-tetrakis[4-(2-trimethylammonioethoxy)-phenyl]ethene
HACD1	3-hydroxyacyl-CoA dehydratase 1

## References

[1] Vafai S., Mootha V. (2012). Mitochondrial disorders as windows into an ancient organelle. Nature.

[2] Pánek T., Eliáš M., Vancová M., Lukeš J., Hashimi H. (2020). Returning to the Fold for Lessons in Mitochondrial Crista Diversity and Evolution. Curr. Biol..

[3] Kondadi A.K., Anand R., Reichert A.S. (2020). Cristae Membrane Dynamics—A Paradigm Change. Trends Cell Biol..

[4] Zick M., Rabl R., Reichert A.S. (2009). Cristae formation—linking ultrastructure and function of mitochondria. Biochim. Biophys. Acta (BBA) Mol. Cell Res..

[5] Cogliati S., Enriquez J.A., Scorrano L. (2016). Mitochondrial Cristae: Where Beauty Meets Functionality. Trends Biochem. Sci..

[6] Colina-Tenorio L., Horten P., Pfanner N., Rampelt H. (2020). Shaping the mitochondrial inner membrane in health and disease. J. Intern. Med..

[7] Glancy B., Kim Y., Katti P., Willingham T.B. (2020). The Functional Impact of Mitochondrial Structure Across Subcellular Scales. Front. Physiol..

[8] Casares D., Escribá P.V., Rosselló C.A. (2019). Membrane Lipid Composition: Effect on Membrane and Organelle Structure, Function and Compartmentalization and Therapeutic Avenues. Int. J. Mol. Sci..

[9] Cullis P., De Kruijff B. (1979). Lipid polymorphism and the functional roles of lipids in biological membranes. Biochim. Biophys. Acta (BBA) Rev. Biomembr..

[10] Shearman G.C., Ces O., Templer R.H., Seddon J.M. (2006). Inverse lyotropic phases of lipids and membrane curvature. J. Phys. Condens. Matter.

[11] Burger K.N. (2000). Greasing Membrane Fusion and Fission Machineries. Traffic.

[12] Frolov V., Shnyrova A., Zimmerberg J. (2011). Lipid Polymorphisms and Membrane Shape. Cold Spring Harb. Perspect. Biol..

[13] Roger A.J., Muñoz-Gómez S.A., Kamikawa R. (2017). The Origin and Diversification of Mitochondria. Curr. Biol..

[14] Raven J.A. (2021). Determinants, and implications, of the shape and size of thylakoids and cristae. J. Plant Physiol..

[15] Frey T.G., Manella C.A. (2000). The internal structure of mitochondria. Trends Biochem. Sci..

[16] Horvath S.E., Daum G. (2013). Lipids of mitochondria. Prog. Lipid Res..

[17] Schlame M. (2021). Protein crowding in the inner mitochondrial membrane. Biochim. Biophys. Acta (BBA) Bioenerg..

[18] Busch K.B. (2020). Inner mitochondrial membrane compartmentalization: Dynamics across scales. Int. J. Biochem. Cell Biol..

[19] Mitchell P. (1961). Coupling of Phosphorylation to Electron and Hydrogen Transfer by a Chemi-Osmotic type of Mechanism. Nature.

[20] Nielsen J., Gejl K., Hey-Mogensen M., Holmberg H.C., Suetta C., Krustrup P., Elemans C., Ortenblad N. (2016). Plasticity in mitochondrial cristae density allows metabolic capacity modulation in human skeletal muscle. J. Physiol..

[21] Acehan D., Xu Y., Stokes D., Schlame M. (2007). Comparison of lymphoblast mitochondria from normal subjects and patients with Barth syndrome using electron microscopic tomography. Lab. Investig. A J. Tech. Methods Pathol..

[22] Siegmund S.E., Grassucci R., Carter S.D., Barca E., Farino Z.J., Juanola-Falgarona M., Zhang P., Tanji K., Hirano M., Schon E.A. (2018). Three-Dimensional Analysis of Mitochondrial Crista Ultrastructure in a Patient with Leigh Syndrome by In Situ Cryoelectron Tomography. iScience.

[23] Prola A., Blondelle J., Vandestienne A., Piquereau J., Denis R.G.P., Guyot S., Chauvin H., Mourier A., Maurer M., Henry C. (2021). Cardiolipin content controls mitochondrial coupling and energetic efficiency in muscle. Sci. Adv..

[24] Palade G.E. (1952). The fine structure of mitochondria. Anat. Rec..

[25] Mannella C.A. (2006). Structure and dynamics of the mitochondrial inner membrane cristae. Biochim. Biophys. Acta (BBA) Mol. Cell Res..

[26] Ikon N., Ryan R.O. (2017). Cardiolipin and mitochondrial cristae organization. Biochim. Biophys. Acta (BBA) Biomembr..

[27] Quintana-Cabrera R., Mehrotra A., Rigoni G., Soriano M. (2018). Who and how in the regulation of mitochondrial cristae shape and function. Biochem. Biophys. Res. Commun..

[28] Blum T.B., Hahn A., Meier T., Davies K.M., Kühlbrandt W. (2019). Dimers of mitochondrial ATP synthase induce membrane curvature and self-assemble into rows. Proc. Natl. Acad. Sci. USA.

[29] Renken C., Siragusa G., Perkins G., Washington L., Nulton J., Salamon P., Frey T.G. (2002). A thermodynamic model describing the nature of the crista junction: A structural motif in the mitochondrion. J. Struct. Biol..

[30] Ponnuswamy A., Nulton J., Mahaffy J.M., Salamon P., Frey T.G., Baljon A.R.C. (2005). Modeling tubular shapes in the inner mitochondrial membrane. Phys. Biol..

[31] Ghochani M., Nulton J., Salamon P., Frey T., Rabinovitch A., Baljon A. (2010). Tensile Forces and Shape Entropy Explain Observed Crista Structure in Mitochondria. Biophys. J..

[32] Gilkerson R.W., Selker J.M., Capaldi R.A. (2003). The cristal membrane of mitochondria is the principal site of oxidative phosphorylation. FEBS Lett..

[33] Vogel F., Bornhövd C., Neupert W., Reichert A.S. (2006). Dynamic subcompartmentalization of the mitochondrial inner membrane. J. Cell Biol..

[34] Song D.H., Park J., Maurer L., Lu W., Philbert M., Sastry A. (2013). Biophysical significance of the inner mitochondrial membrane structure on the electrochemical potential of mitochondria. Phys. Rev. E Stat. Nonlinear Soft Matter Phys..

[35] Westermann B. (2010). Westermann BMitochondrial fusion and fission in cell life and death. Nat. Reviews. Mol. Cell Biol..

[36] Twig G., Hyde B., Shirihai O.S. (2008). Mitochondrial fusion, fission and autophagy as a quality control axis: The bioenergetic view. Biochim. Biophys. Acta (BBA) Bioenerg..

[37] Piquereau J., Caffin F., Novotova M., Prola A., Garnier A., Mateo P., Fortin D., Huynh L.H., Nicolas V., Alavi M.V. (2012). Down-regulation of OPA1 alters mouse mitochondrial morphology, PTP function, and cardiac adaptation to pressure overload. Cardiovasc. Res..

[38] Hackenbrock C.R. (1966). Ultrastructural bases for metabolically linked mechanical activity in mitochondria: I. Reversible Ultrastructural Changes with Change in Metabolic Steady State in Isolated Liver Mitochondria. J. Cell Biol..

[39] Chvanov M. (2006). Metabolic Control of Elastic Properties of the Inner Mitochondrial Membrane. J. Phys. Chem. B.

[40] Almendro-Vedia V.G., Natale P., Mell M., Bonneau S., Monroy F., Joubert F., López-Montero I. (2017). Nonequilibrium fluctuations of lipid membranes by the rotating motor protein F1F0-ATP synthase. Proc. Natl. Acad. Sci. USA.

[41] Huang X., Fan J., Li L., Liu H., Wu R., Wu Y., Wei L., Mao H., Lal A., Xi P. (2018). Fast, long-term, super-resolution imaging with Hessian structured illumination microscopy. Nat. Biotechnol..

[42] Stephan T., Roesch A., Riedel D., Jakobs S. (2019). Live-cell STED nanoscopy of mitochondrial cristae. Sci. Rep..

[43] Wang C., Taki M., Sato Y., Tamura Y., Yaginuma H., Okada Y., Yamaguchi S. (2019). A photostable fluorescent marker for the superresolution live imaging of the dynamic structure of the mitochondrial cristae. Proc. Natl. Acad. Sci. USA.

[44] Wolf D.M., Segawa M., Kondadi A.K., Anand R., Bailey S.T., Reichert A.S., van der Bliek A.M., Shackelford D.B., Liesa M., Shirihai O.S. (2019). Individual cristae within the same mitochondrion display different membrane potentials and are functionally independent. EMBO J..

[45] Segawa M., Wolf D.M., Hultgren N.W., Williams D.S., van der Bliek A.M., Shackelford D.B., Liesa M., Shirihai O.S. (2020). Quantification of cristae architecture reveals time-dependent characteristics of individual mitochondria. Life Sci. Alliance.

[46] Kondadi A.K., Anand R., Hänsch S., Urbach J., Zobel T., Wolf D.M., Segawa M., Liesa M., Shirihai O.S., Weidtkamp-Peters S. (2020). Cristae undergo continuous cycles of membrane remodelling in a MICOS-dependent manner. EMBO Rep..

[47] Osman C., Voelker D.R., Langer T. (2011). Making heads or tails of phospholipids in mitochondria. J. Cell Biol..

[48] Daum G., Vance J.E. (1997). Import of lipids into mitochondria. Prog. Lipid Res..

[49] Mejia E.M., Hatch G.M. (2016). Mitochondrial phospholipids: Role in mitochondrial function. J. Bioenerg. Biomembr..

[50] Kates M., Syz J.Y., Gosser D., Haines T.H. (1993). pH-dissociation characteristics of cardiolipin and its 2′-deoxy analogue. Lipids.

[51] Lewis R.N., McElhaney R.N. (2009). The physicochemical properties of cardiolipin bilayers and cardiolipin-containing lipid membranes. Biochim. Biophys. Acta (BBA) Biomembr..

[52] Sathappa M., Alder N.N. (2016). The ionization properties of cardiolipin and its variants in model bilayers. Biochim. Biophys. Acta (BBA) Biomembr..

[53] Olofsson G., Sparr E. (2013). Ionization Constants pKa of Cardiolipin. PLoS ONE.

[54] Kooijman E., Swim L., Graber Z., Tyurina Y., Bayır H., Kagan V. (2017). Magic angle spinning 31P NMR spectroscopy reveals two essentially identical ionization states for the cardiolipin phosphates in phospholipid liposomes. Biochim. Biophys. Acta (BBA) Biomembr..

[55] Harayama T., Riezman H. (2018). Understanding the diversity of membrane lipid composition. Nat. Rev. Mol. Cell Biol..

[56] Gruner S.M. (1985). Intrinsic curvature hypothesis for biomembrane lipid composition: A role for nonbilayer lipids. Proc. Natl. Acad. Sci. USA.

[57] Oemer G., Koch J., Wohlfarter Y., Alam M.T., Lackner K., Sailer S., Neumann L., Lindner H.H., Watschinger K., Haltmeier M. (2020). Phospholipid Acyl Chain Diversity Controls the Tissue-Specific Assembly of Mitochondrial Cardiolipins. Cell Rep..

[58] Pennington E.R., Funai K., Brown D.A., Shaikh S.R. (2019). The role of cardiolipin concentration and acyl chain composition on mitochondrial inner membrane molecular organization and function. Biochim. Biophys. Acta (BBA) Mol. Cell Biol. Lipids.

[59] Siegel D.P., Epand R.M. (1997). The mechanism of lamellar-to-inverted hexagonal phase transitions in phosphatidylethanolamine: Implications for membrane fusion mechanisms. Biophys. J..

[60] Zimmerberg J. (2006). Membrane biophysics. Curr. Biol..

[61] Kollmitzer B., Heftberger P., Rappolt M., Pabst G. (2013). Monolayer spontaneous curvature of raft-forming membrane lipids. Soft Matter.

[62] Ortiz A., Killian J.A., Verkleij A.J., Wilschut J. (1999). Membrane Fusion and the Lamellar-to-Inverted-Hexagonal Phase Transition in Cardiolipin Vesicle Systems Induced by Divalent Cations. Biophys. J..

[63] Chen Y.F., Tsang K.Y., Chang W.F., Fan Z.A. (2015). Differential dependencies on [Ca2+] and temperature of the monolayer spontaneous curvatures of DOPE, DOPA and cardiolipin: Effects of modulating the strength of the inter-headgroup repulsion. Soft Matter.

[64] Nichols-Smith S., Teh S.Y., Kuhl T. (2004). Thermodynamic and mechanical properties of model mitochondrial membranes. Biochim. Biophys. Acta.

[65] Òscar D., Sanz F., Montero M.T., Hernández-Borrell J. (2006). Thermodynamic and structural study of the main phospholipid components comprising the mitochondrial inner membrane. Biochim. Biophys. Acta (BBA) Biomembr..

[66] Sennato S., Bordi F., Cametti C., Coluzza C., Desideri A., Rufini S. (2005). Evidence of Domain Formation in Cardiolipin—Glycerophospholipid Mixed Monolayers. A Thermodynamic and AFM Study. J. Phys. Chem. B.

[67] Róg T., Martinez-Seara H., Munck N., Orešič M., Karttunen M., Vattulainen I. (2009). Role of Cardiolipins in the Inner Mitochondrial Membrane: Insight Gained through Atom-Scale Simulations. J. Phys. Chem. B.

[68] Dahlberg M., Maliniak A. (2010). Mechanical Properties of Coarse-Grained Bilayers Formed by Cardiolipin and Zwitterionic Lipids. J. Chem. Theory Comput..

[69] Dahlberg M., Maliniak A. (2008). Molecular Dynamics Simulations of Cardiolipin Bilayers. J. Phys. Chem. B.

[70] Khalifat N., Fournier J.B., Angelova M.I., Puff N. (2011). Lipid packing variations induced by pH in cardiolipin-containing bilayers: The driving force for the cristae-like shape instability. Biochim. Biophys. Acta (BBA) Biomembr..

[71] Pöyry S., Róg T., Karttunen M., Vattulainen I. (2009). Mitochondrial Membranes with Mono- and Divalent Salt: Changes Induced by Salt Ions on Structure and Dynamics. J. Phys. Chem. B.

[72] Wilson B.A., Ramanathan A., Lopez C.F. (2019). Cardiolipin-Dependent Properties of Model Mitochondrial Membranes from Molecular Simulations. Biophys. J..

[73] Helfrich W. (1973). Elastic Properties of Lipid Bilayers: Theory and Possible Experiments. Z. FüR Naturforschung.

[74] Unsay J.D., Cosentino K., Subburaj Y., García-Sáez A.J. (2013). Cardiolipin Effects on Membrane Structure and Dynamics. Langmuir.

[75] Pan J., Cheng X., Sharp M., Ho C.S., Khadka N., Katsaras J. (2015). Structural and mechanical properties of cardiolipin lipid bilayers determined using neutron spin echo, small angle neutron and X-ray scattering, and molecular dynamics simulations. Soft Matter.

[76] Boscia A.L., Treece B.W., Mohammadyani D., Klein-Seetharaman J., Braun A.R., Wassenaar T.A., Klösgen B., Tristram-Nagle S. (2014). X-ray structure, thermodynamics, elastic properties and MD simulations of cardiolipin/dimyristoylphosphatidylcholine mixed membranes. Chem. Phys. Lipids.

[77] Rawicz W., Olbrich K., McIntosh T., Needham D., Evans E. (2000). Effect of Chain Length and Unsaturation on Elasticity of Lipid Bilayers. Biophys. J..

[78] Tiberti M., Antonny B., Gautier R. (2020). The transbilayer distribution of polyunsaturated phospholipids determines their facilitating effect on membrane deformation. Soft Matter.

[79] Boyd K., Alder N., May E. (2017). Buckling Under Pressure: Curvature Based Lipid Segregation and Stability Modulation in Cardiolipin Containing Bilayers. Langmuir.

[80] Beltrán-Heredia E., Tsai F.C., Salinas-Almaguer S., Cao F.J., Bassereau P., Monroy F. (2019). Membrane curvature induces cardiolipin sorting. Commun. Biol..

[81] Rand R., Sengupta S. (1972). Cardiolipin forms hexagonal structures with divalent cations. Biochim. Biophys. Acta (BBA) Biomembr..

[82] Cullis P., Verkleij A., Ververgaert P. (1978). Polymorphic phase behaviour of cardiolipin as detected by 31P NMR and freeze-fracture techniques. Effects of calcium, dibucaine and chlorpromazine. Biochim. Biophys. Acta (BBA) Biomembr..

[83] de Kruijff B., Verkleij A., Leunissen-Bijvelt J., van Echteld C., Hille J., Rijnbout H. (1982). Further aspects of the Ca2+-dependent polymorphism of bovine heart cardiolipin. Biochim. Biophys. Acta (BBA) Biomembr..

[84] Fox C.A., Ellison P., Ikon N., Ryan R.O. (2019). Calcium-induced transformation of cardiolipin nanodisks. Biochim. Biophys. Acta (BBA) Biomembr..

[85] Seddon J.M., Kaye R., Marsh D. (1983). Induction of the lamellar-inverted hexagonal phase transition in cardiolipin by protons and monovalent cations. Biochim. Biophys. Acta (BBA) Biomembr..

[86] Khalifat N., Puff N., Bonneau S., Fournier J.B., Angelova M.I. (2008). Membrane Deformation under Local pH Gradient: Mimicking Mitochondrial Cristae Dynamics. Biophys. J..

[87] Khalifat N., Rahimi M., Bitbol A.F., Seigneuret M., Fournier J.B., Puff N., Arroyo M., Angelova M.I. (2014). Interplay of Packing and Flip-flop in Local Bilayer Deformation. How Phosphatidylglycerol Could Rescue Mitochondrial Function in a Cardiolipin-deficient Yeast Mutant. Biophys. J..

[88] Fournier J.B., Khalifat N., Puff N., Angelova M.I. (2009). Chemically Triggered Ejection of Membrane Tubules Controlled by Intermonolayer Friction. Phys. Rev. Lett..

[89] Bitbol A.F., Puff N., Sakuma Y., Imai M., Fournier J.B., Angelova M.I. (2012). Lipid membrane deformation in response to a local pH modification: Theory and experiments. Soft Matter.

[90] Bitbol A.F., Fournier J.B. (2013). Membrane properties revealed by spatiotemporal response to a local inhomogeneity. Biochim. Biophys. Acta (BBA) Biomembr..

[91] Bitbol A.F., Fournier J.B., Angelova M., Puff N. (2011). Dynamical membrane curvature instability controlled by intermonolayer friction. J. Physics. Condens. Matter Inst. Phys. J..

[92] Song D.H., Park J., Philbert M.A., Sastry A.M., Lu W. (2014). Effects of local pH on the formation and regulation of cristae morphologies. Phys. Rev. E Stat. Nonlinear Soft Matter Phys..

[93] Rieger B., Junge W., Busch K.B. (2014). Lateral pH gradient between OXPHOS complex IV and F0F1 ATP-synthase in folded mitochondrial membranes. Nat. Commun..

[94] Patil N., Bonneau S., Joubert F., Bitbol A.F., Berthoumieux H. (2020). Mitochondrial cristae modeled as an out-of-equilibrium membrane driven by a proton field. Phys. Rev. E.

[95] van Meer G., Voelker D.R., Feigenson G.W. (2008). Membrane lipids: Where they are and how they behave. Nat. Rev. Mol. Cell Biol..

[96] Planas-Iglesias J., Dwarakanath H., Mohammadyani D., Yanamala N., Kagan V.E., Klein-Seetharaman J. (2015). Cardiolipin Interactions with Proteins. Biophys. J..

[97] Musatov A., Sedlák E. (2017). Role of cardiolipin in stability of integral membrane proteins. Biochimie.

[98] Beales P.A., Bergstrom C.L., Geerts N., Groves J.T., Vanderlick T.K. (2011). Single Vesicle Observations of the Cardiolipin—Cytochrome c Interaction: Induction of Membrane Morphology Changes. Langmuir.

[99] Bergstrom C.L., Beales P.A., Lv Y., Vanderlick T.K., Groves J.T. (2013). Cytochrome c causes pore formation in cardiolipin-containing membranes. Proc. Natl. Acad. Sci. USA.

[100] Pennington R., Sullivan E., Fix A., Dadoo S., Zeczycki T., DeSantis A., Schlattner U., Coleman R., Chicco A., Brown D. (2018). Proteolipid domains form in biomimetic and cardiac mitochondrial vesicles and are regulated by cardiolipin concentration but not monolyso-cardiolipin. J. Biol. Chem..

[101] Acehan D., Malhotra A., Xu Y., Ren M., Stokes D., Schlame M. (2011). Cardiolipin Affects the Supramolecular Organization of ATP Synthase in Mitochondria. Biophys. J..

[102] Gasanov S.E., Kim A.A., Yaguzhinsky L.S., Dagda R.K. (2018). Non-bilayer structures in mitochondrial membranes regulate ATP synthase activity. Biochim. Biophys. Acta (BBA) Biomembr..

[103] Bazan S., Mileykovskaya E., Mallampalli V., Heacock P., Sparagna G., Dowhan W. (2012). Cardiolipin-dependent Reconstitution of Respiratory Supercomplexes from Purified Saccharomyces cerevisiae Complexes III and IV. J. Biol. Chem..

[104] Szeto H. (2014). First-in-class cardiolipin-protective compound as a therapeutic agent to restore mitochondrial bioenergetics. Br. J. Pharmacol..

[105] Cooke I.R., Deserno M. (2006). Coupling between Lipid Shape and Membrane Curvature. Biophys. J..

[106] Baoukina S., Ingólfsson H., Marrink S., Tieleman D. (2018). Curvature-Induced Sorting of Lipids in Plasma Membrane Tethers. Adv. Theory Simul..

[107] Elías-Wolff F., Lindén M., Lyubartsev A.P., Brandt E.G. (2019). Curvature sensing by cardiolipin in simulated buckled membranes. Soft Matter.

[108] Kamal M.M., Mills D., Grzybek M., Howard J. (2009). Measurement of the membrane curvature preference of phospholipids reveals only weak coupling between lipid shape and leaflet curvature. Proc. Natl. Acad. Sci. USA.

[109] Tian A., Baumgart T. (2009). Sorting of Lipids and Proteins in Membrane Curvature Gradients. Biophys. J..

[110] Sorre B., Callan-Jones A., Manzi J., Goud B., Prost J., Bassereau P., Roux A. (2012). Nature of curvature coupling of amphiphysin with membranes depends on its bound density. Proc. Natl. Acad. Sci. USA.

[111] Prévost C., Zhao H., Manzi J., Lemichez E., Lappalainen P., Callan-Jones A., Bassereau P. (2015). IRSp53 senses negative membrane curvature and phase separates along membrane tubules. Nat. Commun..

[112] Sorre B., Callan-Jones A., Manneville J.B., Nassoy P., Joanny J.F., Prost J., Goud B., Bassereau P. (2009). Curvature-driven lipid sorting needs proximity to a demixing point and is aided by proteins. Proc. Natl. Acad. Sci. USA.

[113] Callan-Jones A., Bassereau P. (2013). Curvature-driven membrane lipid and protein distribution. Curr. Opin. Solid State Mater. Sci..

[114] Krebs J., Hauser H., Carafoli E. (1979). Asymmetric distribution of phospholipids in the inner mitochondrial membrane of beef heart mitochondria. J. Biol. Chem..

[115] Harb J.S., Comte J., Gautheron D.C. (1981). Asymmetrical orientation of phospholipids and their interactions with marker enzymes in pig heart mitochondrial inner membrane. Arch. Biochem. Biophys..

[116] Cheneval D., Müller M., Toni R., Ruetz S., Carafoli E. (1985). Adriamycin as a probe for the transversal distribution of cardiolipin in the inner mitochondrial membrane. J. Biol. Chem..

[117] Petit J.M., Huet O., Gallet P.F., Maftah A., Ratinaud M.H., Julien R. (1994). Direct analysis and significance of cardiolipin transverse distribution in mitochondrial inner membranes. Eur. J. Biochem..

[118] Gallet P.F., Petit J.M., Maftah A., Zachowski A., Julien R. (1997). Asymmetrical distribution of cardiolipin in yeast inner mitochondrial membrane triggered by carbon catabolite repression. Biochem. J..

[119] Gallet P.F., Zachowski A., Julien R., Fellmann P., Devaux P.F., Maftah A. (1999). Transbilayer movement and distribution of spin-labelled phospholipids in the inner mitochondrial membrane. Biochim. Biophys. Acta (BBA) Biomembr..

[120] Jacobson J., Duchen M., Heales S. (2002). Intracellular distribution of the fluorescent dye nonyl acridine orange responds to the mitochondrial membrane potential: Implications for assays of cardiolipin and mitochondrial mass. J. Neurochem..

[121] Gohil V., Gvozdenovic-Jeremic J., Schlame M., Greenberg M. (2005). Binding of 10-N-nonyl acridine orange to cardiolipin-deficient yeast cells: Implications for assay of cardiolipin. Anal. Biochem..

[122] Kagan V., Chu C., Tyurina Y., Cheikhi A., Bayir H. (2013). Cardiolipin asymmetry, oxidation and signaling. Chem. Phys. Lipids.

[123] Schlattner U., Tokarska-Schlattner M., Rousseau D., Boissan M., Mannella C., Epand R., Lacombe M.L. (2014). Mitochondrial cardiolipin/phospholipid trafficking: The role of membrane contact site complexes and lipid transfer proteins. Chem. Phys. Lipids.

[124] Huang K., Mukhopadhyay R., Wingreen N. (2006). A Curvature-Mediated Mechanism for Localization of Lipids to Bacterial Poles. PLoS Comput. Biol..

[125] Elmer-Dixon M.M., Hoody J., Steele H.B.B., Becht D.C., Bowler B.E. (2019). Cardiolipin Preferentially Partitions to the Inner Leaflet of Mixed Lipid Large Unilamellar Vesicles. J. Phys. Chem. B.

[126] Tasseva G., Bai H.D., Davidescu M., Haromy A., Michelakis E., Vance J.E. (2013). Phosphatidylethanolamine Deficiency in Mammalian Mitochondria Impairs Oxidative Phosphorylation and Alters Mitochondrial Morphology*. J. Biol. Chem..

[127] Baker C., Basu Ball W., Pryce E., Gohil V. (2016). Specific Requirements of Non-bilayer Phospholipids in Mitochondrial Respiratory Chain Function and Formation. Mol. Biol. Cell.

[128] van der Veen J., Kennelly J., Wan S., Vance J., Vance D., Jacobs R. (2017). The Critical Role of Phosphatidylcholine and Phosphatidylethanolamine Metabolism in Health and Disease. Biochim. Biophys. Acta (BBA) Biomembr..

[129] Heden T.D., Johnson J.M., Ferrara P.J., Eshima H., Verkerke A.R.P., Wentzler E.J., Siripoksup P., Narowski T.M., Coleman C.B., Lin C.T. (2019). Mitochondrial PE potentiates respiratory enzymes to amplify skeletal muscle aerobic capacity. Sci. Adv..

[130] Bashkirov P.V., Chekashkina K.V., Akimov S.A., Kuzmin P.I., Frolov V.A. (2011). Variation of lipid membrane composition caused by strong bending. Biochem. (Moscow) Suppl. Ser. A Membr. Cell Biol..

[131] Wang H.Y., Bharti D., Levental I. (2020). Membrane Heterogeneity Beyond the Plasma Membrane. Front. Cell Dev. Biol..

[132] Mileykovskaya E., Dowhan W. (2000). Visualization of Phospholipid Domains inEscherichia coli by Using the Cardiolipin-Specific Fluorescent Dye 10-N-Nonyl Acridine Orange. J. Bacteriol..

[133] Koppelman C.M., Den Blaauwen T., Duursma M.C., Heeren R.M.A., Nanninga N. (2001). Escherichia coli Minicell Membranes Are Enriched in Cardiolipin. J. Bacteriol..

[134] Kawai F., Shoda M., Harashima R., Sadaie Y., Hara H., Matsumoto K. (2004). Cardiolipin Domains in Bacillus subtilis Marburg Membranes. J. Bacteriol..

[135] Romantsov T., Helbig S., Culham D.E., Gill C., Stalker L., Wood J.M. (2007). Cardiolipin promotes polar localization of osmosensory transporter ProP in Escherichia coli. Mol. Microbiol..

[136] Mileykovskaya E. (2007). Subcellular localization of Escherichia coli osmosensory transporter ProP: Focus on cardiolipin membrane domains. Mol. Microbiol..

[137] Mukhopadhyay R., Huang K.C., Wingreen N.S. (2008). Lipid Localization in Bacterial Cells through Curvature-Mediated Microphase Separation. Biophys. J..

[138] Renner L.D., Weibel D.B. (2011). Cardiolipin microdomains localize to negatively curved regions of Escherichia coli membranes. Proc. Natl. Acad. Sci. USA.

[139] Jalmar O., García-Sáez A.J., Berland L., Gonzalvez F., Petit P.X. (2010). Giant unilamellar vesicles (GUVs) as a new tool for analysis of caspase-8/Bid-FL complex binding to cardiolipin and its functional activity. Cell Death Dis..

[140] Kawai C., Ferreira J.C., Baptista M.S., Nantes I.L. (2014). Not Only Oxidation of Cardiolipin Affects the Affinity of Cytochrome c for Lipid Bilayers. J. Phys. Chem. B.

[141] Cheniour M., Brewer J., Bagatolli L., Marcillat O., Granjon T. (2017). Evidence of proteolipid domain formation in an inner mitochondrial membrane mimicking model. Biochim. Biophys. Acta (BBA) Gen. Subj..

[142] Tomšiè N., Babnik B., Lombardo D., Mavčič B., Kandušer M., Iglič A., Kralj-Iglič V. (2005). Shape and Size of Giant Unilamellar Phospholipid Vesicles Containing Cardiolipin. J. Chem. Inf. Model..

[143] Domènech O., Redondo L., Picas L., Morros A., Montero M.T., Hernández-Borrell J. (2007). Atomic force microscopy characterization of supported planar bilayers that mimic the mitochondrial inner membrane. J. Mol. Recognit..

[144] Lopes S., Ivanova G., de Castro B., Gameiro P. (2018). Revealing cardiolipins influence in the construction of a significant mitochondrial membrane model. Biochim. Biophys. Acta (BBA) Biomembr..

[145] Jouhet J. (2013). Importance of the hexagonal lipid phase in biological membrane organization. Front. Plant Sci..

[146] Schlame M., Ren M. (2006). Barth syndrome, a human disorder of cardiolipin metabolism. FEBS Lett..

[147] Ohtsuka T., Nishijima M., Suzuki K., Akamatsu Y. (1993). Mitochondrial dysfunction of a cultured Chinese hamster ovary cell mutant deficient in cardiolipin. J. Biol. Chem..

[148] Basu Ball W., Neff J.K., Gohil V.M. (2018). The role of nonbilayer phospholipids in mitochondrial structure and function. FEBS Lett..

[149] Griffiths E.J., Balaska D., Cheng W.H. (2010). The ups and downs of mitochondrial calcium signalling in the heart. Biochim. Biophys. Acta (BBA) Bioenerg..

[150] Mannella C.A. (2020). Consequences of Folding the Mitochondrial Inner Membrane. Front. Physiol..

[151] Heberle J., Riesle J., Thiedemann G., Oesterhelt D., Dencher N.A. (1994). Proton migration along the membrane surface and retarded surface to bulk transfer. Nature.

[152] Ferguson S.J. (1995). Chemiosmotic Coupling: Protons fast and slow. Curr. Biol..

[153] Yoshinaga M.Y., Kellermann M.Y., Valentine D.L., Valentine R.C. (2016). Phospholipids and glycolipids mediate proton containment and circulation along the surface of energy-transducing membranes. Prog. Lipid Res..

[154] Morelli A., Ravera S., Calzia D., Panfoli I. (2019). An update of the chemiosmotic theory as suggested by possible proton currents inside the coupling membrane. Open Biol..

[155] Toth A., Meyrat A., Stoldt S., Santiago R., Wenzel D., Jakobs S., von Ballmoos C., Ott M. (2020). Kinetic coupling of the respiratory chain with ATP synthase, but not proton gradients, drives ATP production in cristae membranes. Proc. Natl. Acad. Sci. USA.

[156] Haines T.H., Dencher N.A. (2002). Cardiolipin: A proton trap for oxidative phosphorylation. FEBS Lett..

